# *Aeromonas* spp. as a fast-growing high-performance chassis for protein production

**DOI:** 10.1128/aem.00780-25

**Published:** 2025-06-03

**Authors:** Ming-Xuan Tang, Peng-Fei Meng, Ruo-Lin Huang, Xin Zheng, Chen-Chen Liang, Xuepiao Pu, Chen Wang, Ying Zhao, Yi-Qiu Zhang, Jia-Xin Liang, Yu-Xi Yan, Yanyu Xiao, Ying An, Xiaoye Liang, Yi Song, Jiuxin Qu, Bo Yu, Yu Xia, Tao Dong

**Affiliations:** 1 School of Life Sciences, Guangming Advanced Research Institute, Southern University of Science and Technology 656360 , Shenzhen, Guangdong, China; 2 School of Environmental Science and Engineering, Southern University of Science and Technology 726389 , Shenzhen, Guangdong, China; 3 Department of Clinical Laboratory, Shenzhen Third People’s Hospital, National Clinical Research Center for Infectious Diseases, The Second Affiliated Hospital of Southern University of Science and Technology 535206 , Shenzhen, Guangdong, China; 4 Department of Microbial Physiological & Metabolic Engineering, State Key Laboratory of Microbial Diversity and Innovative Utilization, Institute of Microbiology, Chinese Academy of Sciences, Beijing, China; Indiana University Bloomington, Bloomington, Indiana, USA

**Keywords:** protein expression, resist phage infection, transcriptome, OMVs

## Abstract

Recombinant protein production is crucial for biotechnology and industrial processes. While *Escherichia coli* and other bacterial systems are effective, alternative systems can complement their limitations in specific applications. We introduce AMAX, a fast-growing high-performance bacterial chassis with target protein yields comprising 60–70% of total protein content. AMAX is compatible with common protein expression vectors, exhibits a growth rate comparable to *Vibrio natriegens*, and can adapt to diverse conditions, including co-production with *E. coli*, freshwater to seawater salinity, and contaminant phages. We also demonstrate the versatility of AMAX in producing several commercially valuable enzymes at high yield and purity. Transcriptomic and proteomic analyses highlight its robust regulatory networks and potential for outer membrane vesicle (OMV)-mediated cargo delivery. Safety evaluation using multiple eukaryotic models indicates it is nontoxic. These results demonstrate AMAX as a valuable tool for recombinant protein production.

**IMPORTANCE:**

AMAX complements current systems by addressing challenges such as phage contamination and high GC-content protein expression, while offering rapid growth, high protein yields, and adaptability to saline environments. Its favorable biosafety profile and potential for OMV-based protein delivery further enhance its application, making it a versatile platform for sustainable and efficient bioproduction.

## INTRODUCTION

The production of recombinant proteins is a cornerstone of modern biotechnology. These proteins have diverse applications, including life-saving therapies, efficient bioprocesses, and groundbreaking discoveries. Currently, several platforms are employed for recombinant protein production, including prokaryotic systems like *Escherichia coli*, or yeast, insect cells, and mammalian systems (1). Among these, *E. coli* is widely used for its well-characterized genetics, rapid growth, and cost-effective cultivation. However, many proteins fail to be expressed efficiently in *E. coli* due to codon usage bias, toxicity, poor solubility, and folding difficulties (2–4). Alternative microbial platforms are needed to address these challenges, especially for production under nonstandard conditions. Known chassis, such as *Vibrio natriegens* (5, 6), *Pseudomonas putida* (7–9), and *Bacillus subtilis* (10–12), expand the toolbox for diverse biotechnological applications. As an emerging chassis, *V. natriegens* has shown great potential due to its exceptionally rapid doubling times and high biomass yields. However, its low GC (guanine-cytosine) content, dependence on specialized media, and requirement for optimized genetic tools underscore the need for complementary expression systems.

To ensure the safe application of protein-producing chassis systems, genome-level analyses, particularly those of virulence factors and antibiotic resistance, provide a more reliable assessment than species designation alone. For example, despite the existence of many pathogenic *E. coli* strains (13), nonpathogenic laboratory strains, such as BL21 and DH5α, are widely used as safe and reliable tools for biotechnology. Additionally, pathogenic bacteria have been harnessed for beneficial applications when appropriately modified or attenuated. For instance, attenuated *Salmonella* (14–16) and *Mycobacterium bovis* (17–20) strains have been successfully employed as live vaccines, demonstrating how genetic modifications can mitigate safety concerns while leveraging advantageous traits.

*Aeromonas* species represent another promising resource for biotechnology due to their ubiquity in aquatic and terrestrial environments, rapid growth rates, and metabolic adaptability (21–25). *Aeromonas* species are Gram-negative, facultatively anaerobic bacilli that thrive in diverse environmental stress conditions, such as salinity fluctuations, temperature changes, and nutrient limitations (21, 22). The genus includes over 36 recognized species (22), exhibiting versatile metabolic capabilities, such as degrading complex organic compounds and producing extracellular enzymes. Only a small subset of *Aeromonas* species and strains are known to be pathogenic, with four species (*A. caviae*, *A. dhakensis*, *A. veronii*, and *A. hydrophila*) accounting for over 95% of reported clinical cases (25).

In this study, we present AMAX, a novel protein production chassis derived from *Aeromonas*. AMAX combines rapid growth and significantly improved protein production efficiency relative to *E. coli* and *V. natriegens* cells, with target proteins constituting 60–70% of the total cellular protein content. It is compatible with common expression vectors and offers high protein yields even when co-cultured with *E. coli* in the presence of *E. coli* phages. Transcriptomic analyses reveal its robust transcription, translation, metabolism, and regulatory pathways during the transition from logarithmic to stationary growth phases. Proteomic profiling of its outer membrane vesicles (OMVs) identified two candidate proteins, Pal and K1JB12, capable of mediating the delivery of target cargo proteins into OMVs. Importantly, biosafety evaluations using HeLa cells, amoeba, and *Caenorhabditis elegans* detected no toxicity. We demonstrate that AMAX efficiently produces multiple commercial enzymes (T4 DNA ligase, T7 RNA polymerase, Ulp1 protease [26], HRV 3C protease [27], and D-hydantoinase [28]) with high yields and purity. These data collectively support that AMAX exhibits great potential as a versatile tool for scalable recombinant protein production.

## RESULTS

### Genomic insights and protein production potential of AMAX

**Fig 1:**
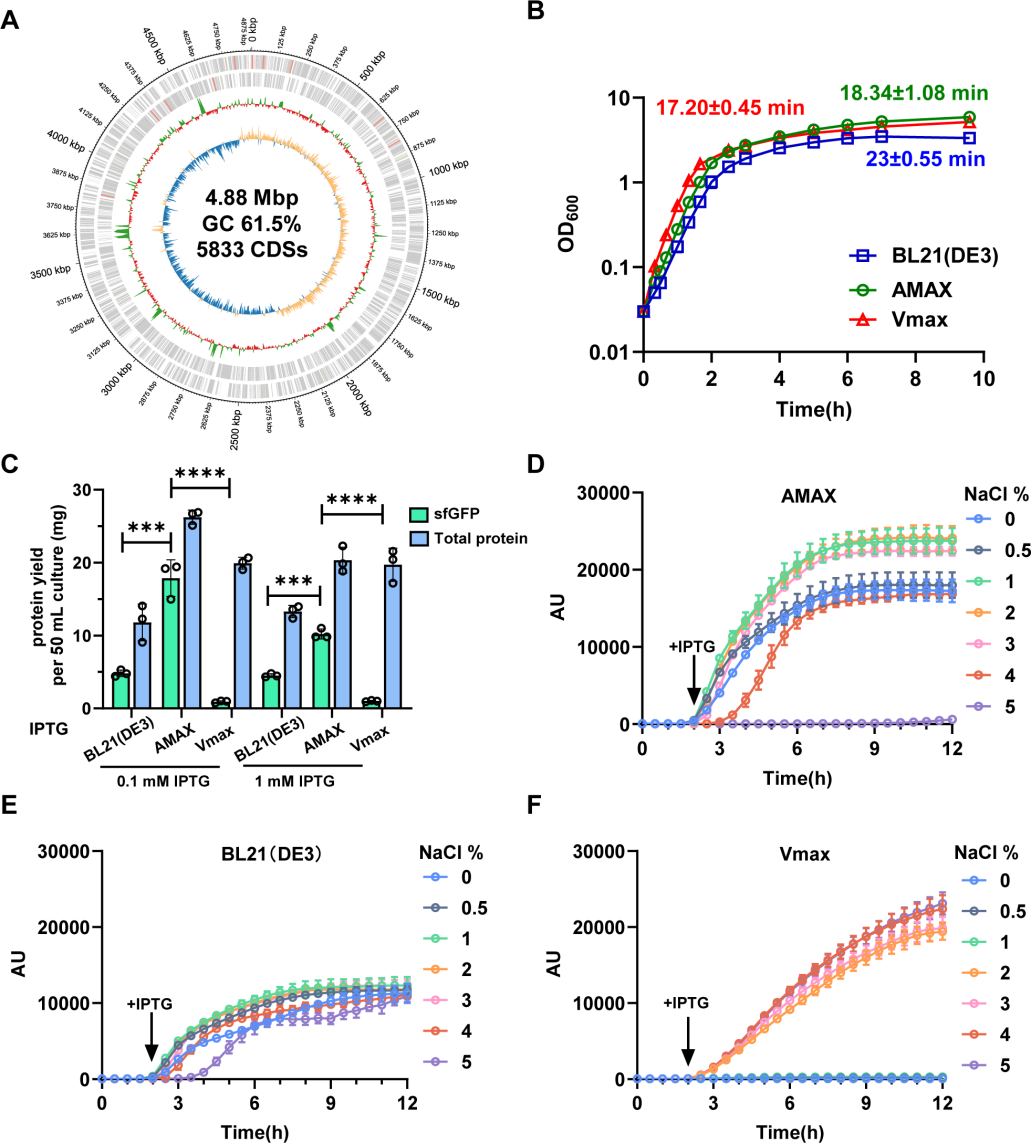
AMAX as a tool for rapid growth and superior protein expression. (**A**) Schematic representation of the genome characteristics. From the outermost to the innermost circles: Genome scale—the outermost circle represents the genome’s positional scale. Genes on the + strand: the second circle depicts genes encoded on the positive (+) strand, with rRNA genes highlighted in orange. Genes on the − strand: the third circle illustrates genes encoded on the negative (−) strand. GC content: the fourth circle shows the GC content, where regions with GC content higher than the genome-wide average are marked in green, and regions with lower GC content are marked in red. GC skew: the innermost circle displays the GC skew. Regions where G content exceeds C content are shown in orange, while regions with higher C content than G content are shown in blue. (**B**) Comparison of growth curves of AMAX, BL21(DE3), and Vmax. (**C**) Comparison of the total protein and sfGFP yield of AMAX, BL21(DE3), and Vmax. (D–F) Fluorescence intensity of sfGFP produced by AMAX (**D**), BL21(DE3) (**E**), and Vmax (**F**) in LB medium under different NaCl concentrations, determined using a 96-well microplate reader. For C, error bars indicate the mean ± standard deviation of three biological replicates. Statistical significance was calculated using ordinary one-way ANOVA with Tukey’s multiple comparison test. ***, *P* < 0.001; ****, *P* < 0.0001. AU, arbitrary units.

To initiate our investigation, we selected a rapidly growing *Aeromonas* isolate from our environmental laboratory collection, which demonstrated fast growth in the LB medium. This strain was genetically modified to incorporate a T7-inducible expression system at the locus between *sprT* and *dns* genes of the genome (29), making it suitable for the application of pET expression vectors. In addition, we carefully removed potential virulence genes and secretion systems (T3SS and T6SS) to ensure safety (Table S1). The resulting strain, named AMAX, was analyzed for its genomic features and protein production capabilities. Using nanopore sequencing and Prokka annotation (30), we found that the AMAX genome spans 4.88 Mb, has a GC content of 61.5%, and encodes 5,833 coding sequences, including 127 tRNAs, one tmRNA, and 31 rRNAs, resulting in a coding density of 84.6% (Fig. 1A). Genome analysis confirms the lack of known virulence genes in AMAX and its genetic relation with other *Aeromonas* species.

For introducing plasmids, we found that AMAX is compatible with standard electroporation, achieving transformation efficiencies of 10⁶–10⁷ CFU/μg with pUC19. We demonstrated that AMAX stably maintains plasmids with various origins of replication—p15A (*p15A vector*), pMB1 (*pET vectors*), and pMB1-derived pBR322 (*pBAD* and *pPSV vectors*)—as well as resistance cassettes for ampicillin, kanamycin, chloramphenicol, and gentamicin. In addition to the T7 promoter, the arabinose-inducible *Para*, anhydrotetracycline-inducible *Ptet*, and IPTG-inducible *PlacUV5* promoters could effectively drive gene expression in AMAX (Table S2).

We compared the growth rate of AMAX with two widely studied bacterial strains: *E. coli* BL21(DE3) and *V. natriegens* (Vmax). In shaking flask LB cultures, AMAX exhibited a doubling time of 18.34 ± 1.08 minutes, outperforming BL21(DE3) (23.00 ± 0.55 minutes) and approaching the rapid growth of Vmax (17.20 ± 0.45 minutes in LB with 3% NaCl) under routine laboratory conditions (Fig. 1B).

Next, we evaluated protein production capacity using sfGFP as a model protein (Fig. S1A). The total protein yield was measured using the BCA (bicinchoninic acid) protein assay (31), with BSA (bovine serum albumin) as the standard (Fig. S1B), while the sfGFP yield was determined based on fluorescence intensity, using purified sfGFP as the standard (Fig. S1C and D). Induction with 0.1 mM IPTG at 37°C for 3 hours yielded approximately 15–20 mg of sfGFP per 50 mL of fermentation medium in AMAX, approximately three times higher than the yield from BL21(DE3) (Fig. 1C). Notably, sfGFP accounted for 60–70% of the total cellular protein in AMAX, underscoring its exceptional protein production capability. Unlike Vmax and BL21(DE3), AMAX also exhibited a sharp increase in protein production following induction, suggesting a faster production rate than the other two chassis cells.

Next, we investigated the salinity tolerance of AMAX compared to BL21(DE3) and Vmax. AMAX demonstrated optimal growth and protein expression within a salt concentration range of 1% to 3%, with growth arrested at 5% salinity (Fig. 1D; Fig. S1E). This range aligns well with seawater salinity (~3%), making AMAX suitable for seawater fermentation processes. By contrast, BL21(DE3) expressed sfGFP at all salinity levels but significantly less than AMAX (Fig. 1E; Fig. S1F). Vmax exhibited AMAX-comparable production at salt levels between 2% and 5% and could not grow below 1% salinity (Fig. 1F; Fig. S1G). Collectively, these results suggest that AMAX is a fast-growing and versatile chassis with exceptional protein production capabilities.

### Mixed-strain fermentation safeguards against phage contamination

**Fig 2:**
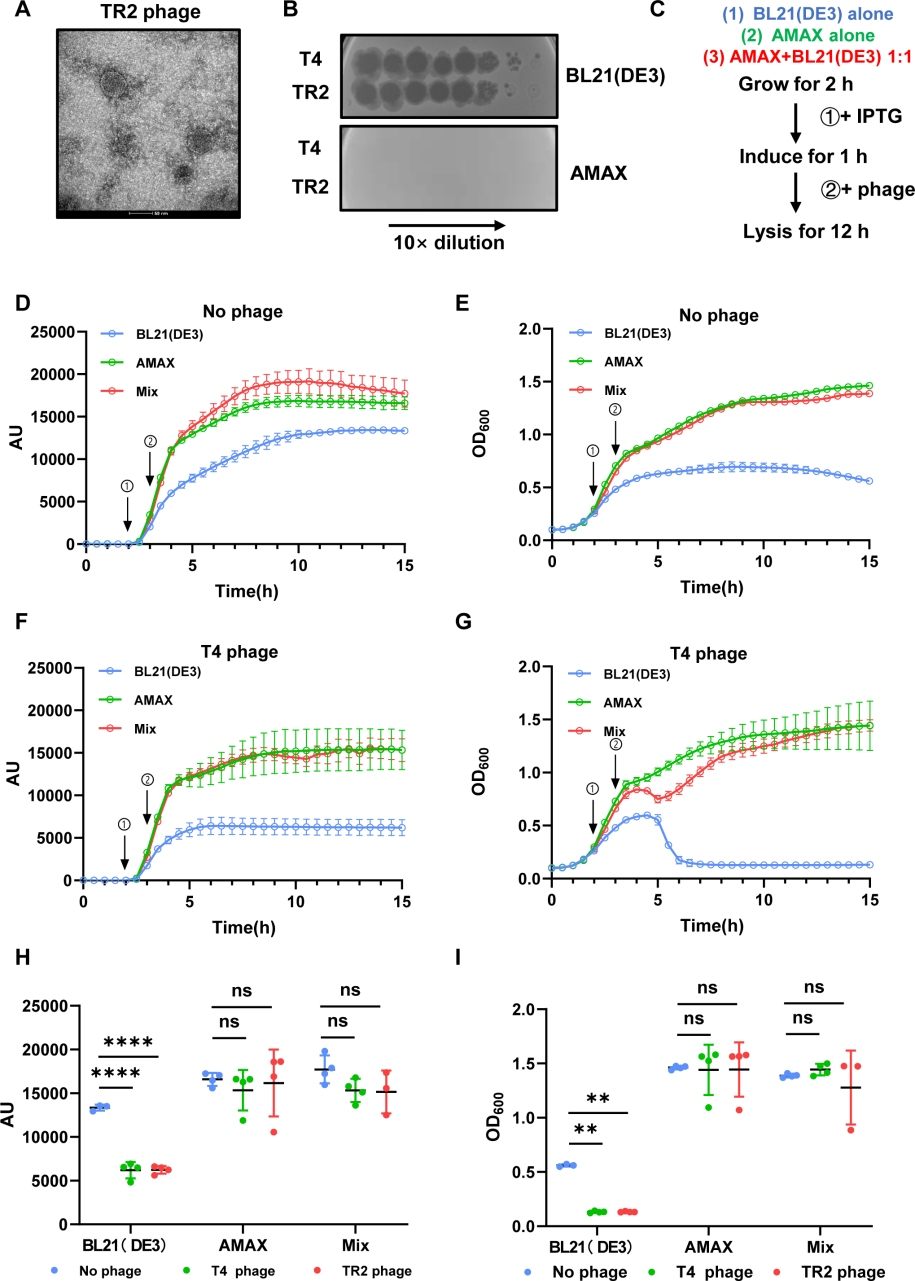
Mixed-strain fermentation provides protection against phage contamination. (**A**) Transmission electron microscopy (120 kV) image of the isolated TR2 phage. (**B**) Plaque assay results for BL21(DE3) and AMAX infected with *E. coli* T4 and TR2 phages. (**C**) Workflow for evaluating the resistance of three systems to *E. coli* phage contamination. (D and E) Protein expression (**D**) and growth (**E**) of the three systems in the absence of phage perturbation. (F and G) Protein expression (**F**) and growth (**G**) of the three systems upon T4 phage infection. (H and I) Final sfGFP (**H**) and OD_600_ yield (**I**) of BL21(DE3) alone, AMAX alone, and a 1:1 mixed fermentation system of BL21(DE3) and AMAX, with and without infection by T4 or TR2 phages, as determined using a 96-well microplate reader. Error bars indicate the mean ± standard deviation of at least three biological replicates. Statistical significance was calculated using two-way ANOVA with Dunnett’s multiple comparison test. ns, not significant; **, *P* < 0.01; ****, *P* < 0.0001. AU, arbitrary units.

Bacteriophage contamination remains a critical challenge in industrial fermentation, leading to reduced yields and significant economic losses (32–36). To address this, we evaluated the susceptibility of AMAX to *E. coli* phages, including T4 and an environmentally isolated Caudovirales phage with sequence similarity to TR2 (Fig. 2A). Plaque assays confirmed that AMAX is resistant to both phages, unlike BL21(DE3), which was rapidly lysed (Fig. 2B). This resistance aligns with the host range specificity of bacteriophages, highlighting AMAX as a robust alternative in phage-prone fermentation environments.

Industrial and laboratory protein production systems typically rely on a single strain, which makes them highly susceptible to phage infections. To mitigate this vulnerability, we tested a mixed-strain production strategy, combining AMAX and BL21(DE3) in a 1:1 ratio. The rationale is to reduce the risk of phage-induced lysis, as phages targeting one strain would not affect the other due to host specificity. However, for this approach to be effective, it is crucial that the two strains are compatible and do not exhibit antagonistic effects on their growth or protein production. This mixture was tested against T4 and TR2 phages alongside single-strain samples of BL21(DE3) and AMAX (Fig. 2C). In the absence of phages, all samples exhibited robust growth and sfGFP production (Fig. 2D and E). However, under phage exposure, BL21(DE3)-only systems experienced rapid lysis, leading to a cessation of sfGFP production. In contrast, AMAX-only systems remained unaffected due to phage resistance (Fig. 2F and G; Fig. S2A and B). In the mixed sample, initial phage-induced lysis of BL21(DE3) resulted in a temporary decline in OD_600_, but AMAX growth subsequently dominated, restoring biomass and sfGFP production to levels comparable with AMAX-only samples (Fig. 2H and I). This demonstrates that mixed-strain production, involving the same expression vectors expressed in AMAX and BL21(DE3) chassis cells, can mitigate the risks of phage contamination while maintaining productivity.

### Transcriptome analysis of AMAX in exponential and stationary phases

**Fig 3:**
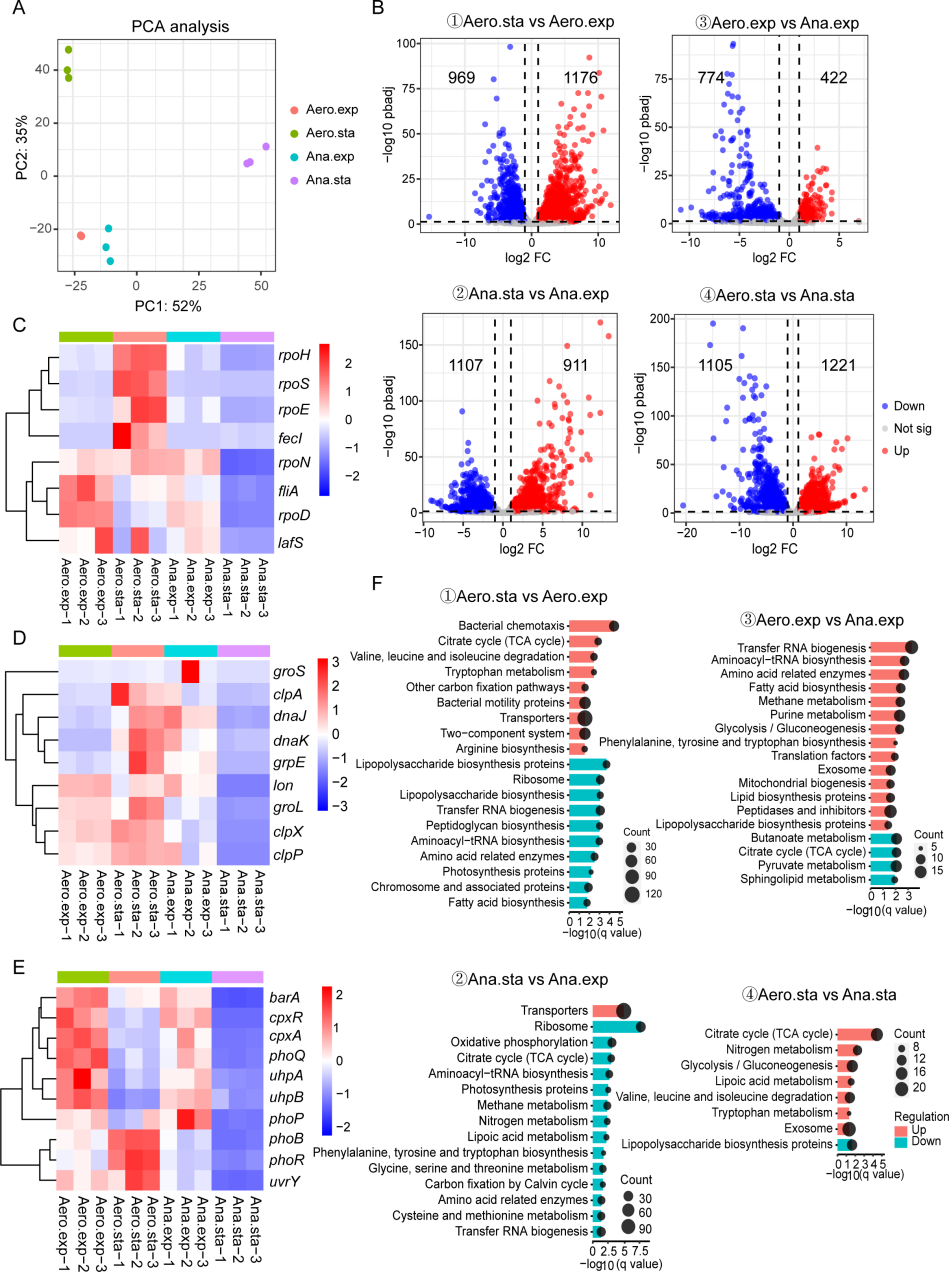
Transcriptome analysis of AMAX during exponential and stationary phases. (**A**) PCA plot depicting the correlation among samples. The x-axis represents the first principal component (PC1), and the y-axis represents the second principal component (PC2). The percentage of variance explained by each component is shown in parentheses. (**B**) Volcano plot illustrating the differentially expressed genes across four comparisons, with upregulated genes shown in red, downregulated genes in blue, and genes without significant regulation in gray. (C–E) Heatmap showing the expression levels of sigma factors (**C**), chaperones and proteases (**D**), and TCSs (**E**) in each sample. The scale represents normalized TPM values by *Z*-score. (**F**) KEGG enrichment analysis of differentially expressed genes across four comparisons, with enriched pathways having a *q*-value ≤0.05 shown.

Understanding the transcriptome changes during growth is crucial for optimizing its use as a chassis. We collected RNA samples during logarithmic and late stationary phases under aerobic and anaerobic conditions (denoted as Aero.exp, Aero.sta, Ana.exp, and Ana.sta, respectively). Transcriptomes were analyzed using principal component analysis (PCA), which demonstrated robust clustering and reproducibility (Fig. 3A). By applying a threshold of |log_2_ Fold change| ≥ 1 and log_10_ pbadj ≤0.05, we identified the differentially expressed genes (DEGs) across the four comparisons among the twelve samples: (1) Aero.sta vs. Aero.exp, (2) Ana.sta vs. Ana.exp, (3) Aero.exp vs. Ana.exp, and (4) Aero.sta vs. Ana.sta (Fig. 3B). Overall, about 80% genes were expressed in each phase, with ~40% of genes showing changes in comparison to (1) Aero.sta vs. Aero.exp, (2) Ana.sta vs. Ana.exp, and (4) Aero.sta vs. Ana.sta, and ~20% of genes showing changes in comparison to (3) Aero.exp vs. Ana.exp, indicating that AMAX possesses an effective regulatory network that controls substantial transcription reprogramming during growth changes.

We first focused on the changes in transcription, translation, regulation, and metabolism between the exponential phase and the stationary phase. Because bacterial transcription regulation primarily relies on the sigma subunits of RNA polymerase, we compared the expression of eight known sigma factors across the four growth phases, which exhibited phase-specific expression patterns (Fig. 3C). Under aerobic conditions, *rpoD* and *filA* were predominant during exponential growth, while *rpoS*, *rpoH*, *rpoE*, and *fecI* were upregulated in the stationary phase. Anaerobic conditions showed a similar trend, with *rpoD* and *rpoN* highly expressed during exponential growth and a general decline in sigma factor expression during the stationary phase (Fig. 3C). These patterns align with known observations in *E. coli*, where RpoD is the essential housekeeping sigma factor required for exponential-phase growth and RpoS is the stationary-phase sigma factor responsible for stationary-phase gene expression. We also noticed increased expression of chaperones (e.g., *dnaJ-dnaK-grpE*) and proteases (e.g., *clpA*) during the stationary phase, indicating elevated protein-folding stress (Fig. 3D).

AMAX is predicted to contain 17 distinct two-component systems (TCSs) that regulate various cellular processes in response to environmental cues (Table S3) (37). Under aerobic conditions, the expression of the glucose-6-phosphate-responsive UhpBA system was higher during the exponential phase (Fig. 3E). In contrast, the PhoRB system, which is activated under phosphate starvation, was upregulated during the stationary phase, indicating an adaptive response to nutrient limitation. Under anaerobic conditions, we observed increased expression of the PhoPQ, CpxAR, and BarA-UvrY systems during the exponential phase. The PhoPQ system responds to magnesium deficiency, while CpxAR is involved in extracellular stress response pathways. The BarA-UvrY system, which regulates the central carbon metabolism, plays a critical role in bacterial adaptation to nutrient limitation and other stress conditions. We further analyzed the potential role of the ppGpp-mediated stringent response during growth-phase transitions. Notably, the expression levels of RelA and SpoT showed no significant differences between stationary and exponential phases under either aerobic or anaerobic conditions, suggesting that stringent control may not play a major role in this regulatory process.

Metabolic shifts were further highlighted by KEGG (Kyoto Encyclopedia of Genes and Genomes) (38) enrichment analysis of DEGs. During the transition to the stationary phase, upregulated pathways included bacterial chemotaxis, the TCA cycle, and amino acid degradation, while lipopolysaccharide and fatty acid biosynthesis pathways were downregulated (Fig. 3F). These changes indicate the ability of AMAX to reprogram metabolic activities to optimize survival under nutrient-limited conditions.

### Transcription analysis of AMAX under aerobic and anaerobic conditions

Oxygen availability significantly influences fermentation outcomes, affecting microbial growth, product quality, and yield (39). Comparing transcriptional profiles under aerobic and anaerobic conditions revealed notable stress adaptations of AMAX. While sigma factors and two-component systems (TCSs) showed no major changes in expression during exponential growth (Aero.exp vs. Ana.exp, Fig. 3C and E), the *dnaJ-dnaK-grpE* operon was significantly upregulated under anaerobic conditions, indicating elevated protein-folding stress (Fig. 3D). During the stationary phase (Aero.sta vs. Ana.sta), the overall expression of sigma factors, proteases, chaperones, and TCSs declined, suggesting that AMAX transitioned to a growth-limiting state (Fig. 3C and E). KEGG enrichment analysis revealed that pathways, such as glycolysis/gluconeogenesis and exosome-related functions, were upregulated under aerobic conditions and suppressed under anaerobic conditions. Conversely, the TCA cycle exhibited differential regulation: it was downregulated during exponential growth but upregulated during the stationary phase under anaerobic conditions (Fig. 3F). These findings provide insights into its robustness and potential optimization for anaerobic fermentation processes.

### Proteome analysis of OMVs derived from AMAX

**Fig 4:**
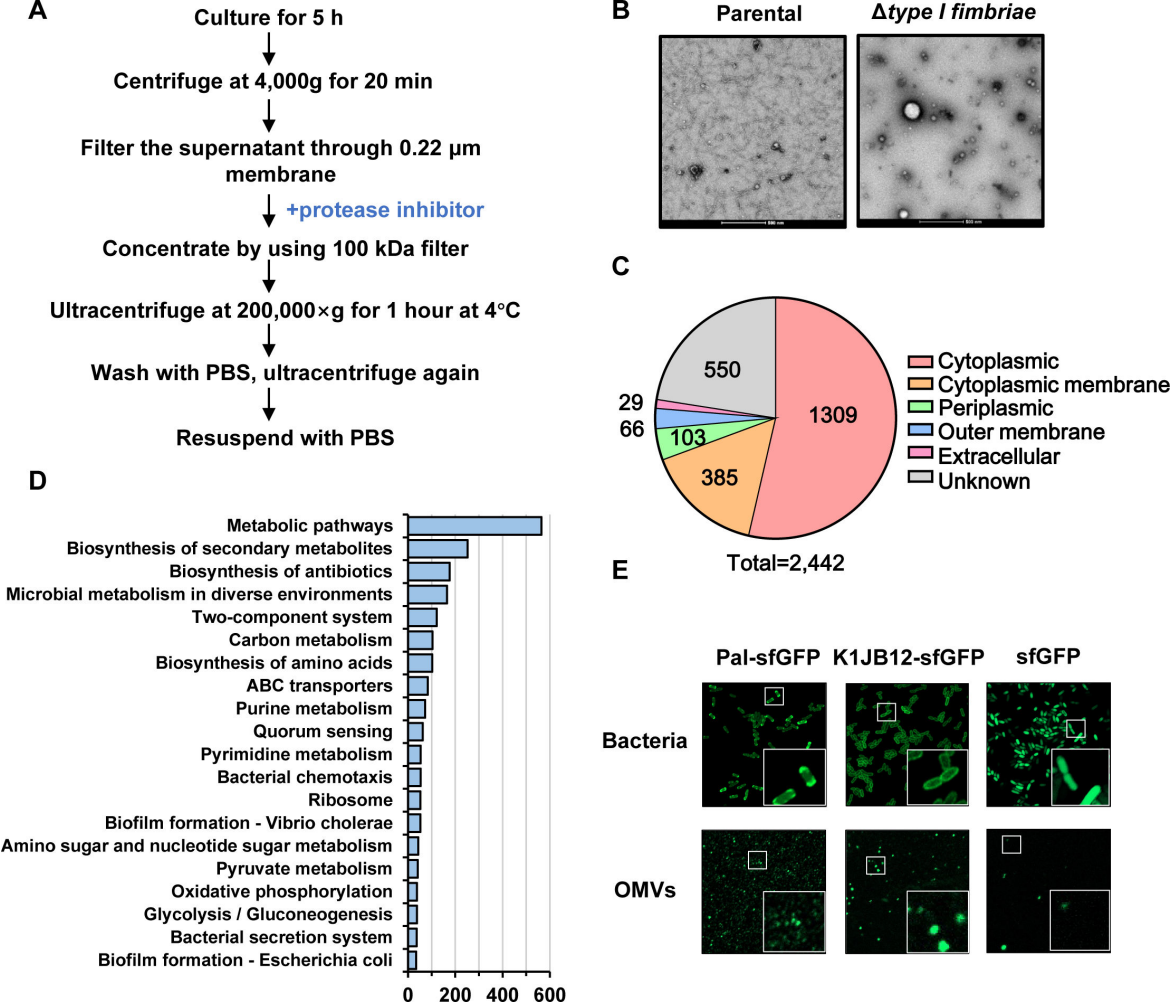
Proteome analysis of OMVs derived from AMAX. (**A**) A detailed workflow illustrating the extraction process of OMVs. (**B**) Transmission electron microscopy images depicting the OMVs isolated from AMAX and its Δ*type I fimbriae* mutant. (**C**) A pie chart representing the subcellular localization of proteins identified within the OMVs. (**D**) KEGG enrichment analysis of the proteins present in OMVs, highlighting the top 20 enriched pathways. (**E**) Fluorescence microscopy images showcasing bacteria and OMVs extracted from AMAX overexpressing sfGFP, Pal-sfGFP, or K1JB12-sfGFP. A representative 30 × 30 µm field of cells is shown, with a 3× magnified 5 × 5 µm inset (marked by box).

Outer membrane vesicles (OMVs) are nanoscale structures produced by bacteria, typically ranging from 20 to 400 nm in diameter, which facilitate critical biological functions, such as quorum sensing, horizontal gene transfer, nutrient transport, and interactions with eukaryotic hosts (40–42). To investigate the composition and functionality of OMVs in AMAX, we isolated OMVs from a strain with the type I fimbriae system knocked out, minimizing contamination during extraction (Fig. 4A and B).

Label-free DIA quantitative proteomics identified 2,442 proteins in OMVs across three replicates, with 2,327 proteins consistently detected (Fig. S3A). Subcellular localization analysis using PSORTb v3.0 (43) revealed that OMVs predominantly contained cytoplasmic (1,309 proteins), inner membrane (385 proteins), periplasmic (103 proteins), outer membrane (66 proteins), and extracellular proteins (29 proteins), along with 550 proteins of unknown localization (Fig. 4C). Notably, the two most abundant proteins were prophage-associated: K1JNX6, a bacteriophage Mu GpT domain-containing protein, and K1JH43, a major capsid protein from the p2 family of phages. Other key proteins included ExeM, a critical enzyme for biofilm development via extracellular DNA degradation, the molecular chaperone GroL, and three outer membrane proteins (OmpK, OmpC, and OmpA) (Fig. S3B). As expected, no known toxin was detected.

Gene ontology (GO) enrichment analysis of OMV proteins highlighted significant involvement in processes such as oxidation-reduction reactions, transcriptional regulation, proteolysis, phosphorylation, and transport. Membrane-associated functions dominated the cellular component (CC) module, while ATP binding, DNA/RNA binding, and ion binding were prominent in the molecular function (MF) module (Fig. S3C). KEGG pathway analysis further revealed enrichment in pathways linked to secondary metabolite and antibiotic biosynthesis, two-component systems, quorum sensing, chemotaxis, and biofilm formation (Fig. 4D).

To test whether cargo proteins can be loaded to OMV, we fused sfGFP to the C-termini of these proteins. Microscopic visualization revealed that Pal-sfGFP and K1JB12-sfGFP localize in the bacteria membrane and are efficiently transported into OMVs (Fig. 4E). In contrast, sfGFP alone was unable to be recruited into the OMVs derived from AMAX.

### AMAX is susceptible to antibiotics and nontoxic to eukaryotic models

**Fig 5:**
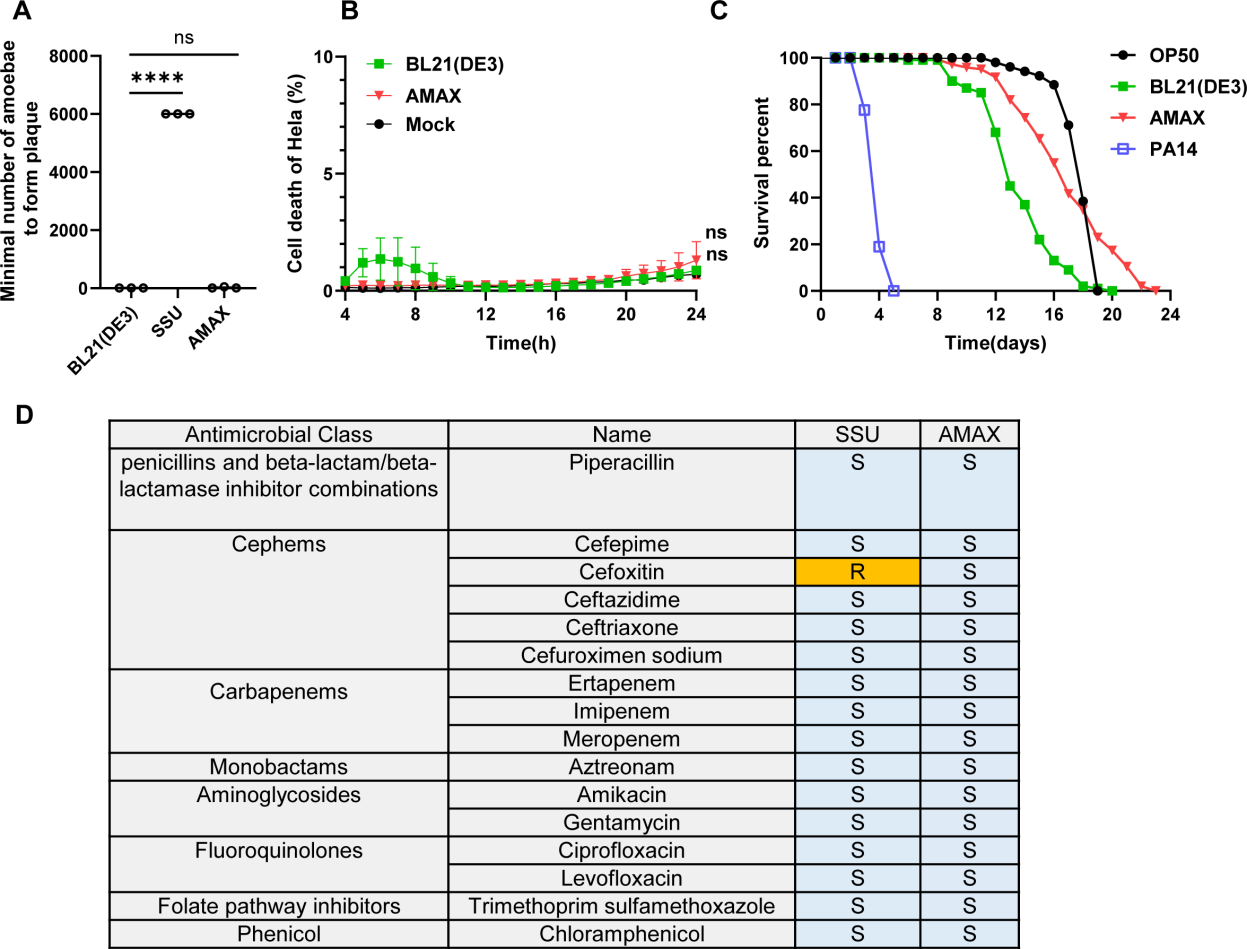
Biosafety assessment of AMAX. (**A**) Competition between BL21(DE3), SSU, and AMAX with phagocytic amoeba. (**B**) Cell viability of HeLa cells following infection with various bacteria at an MOI of 20, assessed by PI staining of dead cells. (**C**) Survival curve of nematodes infected with different bacterial strains. (**D**) Antimicrobial resistance profile of AMAX. For A and B, statistical significance was calculated using two-way ANOVA with Tukey’s multiple comparison test. ns, not significant; ****, *P* < 0.0001.

The safety of AMAX was evaluated using three eukaryotic models: *Dictyostelium discoideum* amoebae, HeLa epithelial cells, and *C. elegans*. In a Dicty competition assay, a known pathogen, *Aeromonas dhakensis* SSU, significantly inhibited amoeba growth. In contrast, AMAX caused minimal inhibition similar to BL21(DE3) (Fig. 5A). In HeLa cells, propidium iodide (PI) staining revealed negligible cytotoxicity from AMAX, comparable to BL21(DE3) and mock controls (Fig. 5B; Fig. S4). In contrast, SSU induced rapid cell rounding, indicative of cytotoxicity (Fig. S4). In the nematode model, infections with AMAX caused no significant mortality, with nematodes exhibiting normal growth and development, often outperforming those exposed to the nonpathogenic BL21(DE3) strain (Fig. 5C). In contrast, the control strain *Pseudomonas aeruginosa* PA14 was highly toxic to worms (44).

Antibiotic susceptibility testing, following the Clinical and Laboratory Standards Institute (CLSI) guidelines, confirmed that AMAX strains were sensitive to all tested antimicrobials, including penicillins, cephems, carbapenems, monobactams, aminoglycosides, fluoroquinolones, folate pathway inhibitors, and phenicols. By comparison, SSU displayed resistance to cefoxitin (Fig. 5D).

Collectively, these results indicate that AMAX is a safe protein production platform with minimal cytotoxicity across multiple eukaryotic models and broad sensitivity to clinically relevant antibiotics.

### AMAX enables rapid and versatile production of commercial enzymes

**Fig 6:**
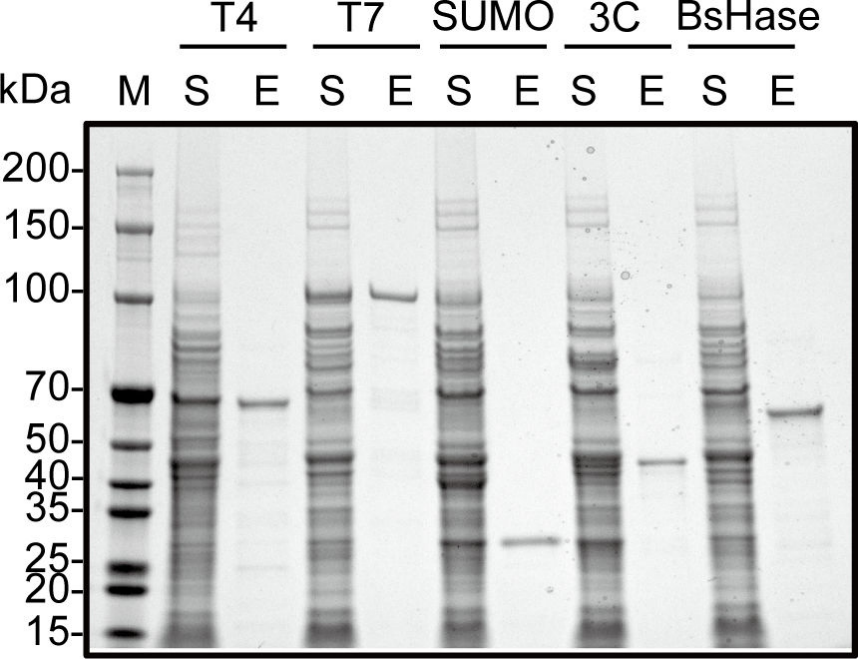
SDS-PAGE analysis of purified enzymes in AMAX. Enzymes (T4 ligase, T7 RNAP, SUMO protease, 3C protease, and BsHase) were expressed in AMAX by induction with 0.1 mM IPTG at 37°C for 3 hours. Cell pellets were resuspended in lysis buffer and lysed using high-pressure homogenization. The lysates were clarified by high-speed centrifugation, and the supernatant fractions were used for affinity purification. Proteins were purified using either Ni–NTA or glutathione agarose beads, depending on the tag (His or GST). Elution was performed with imidazole or glutathione gradients. The supernatant (S) and elution (E) fractions for each enzyme were subject to SDS-PAGE analysis. The quantification of the purified protein was determined using a modified BCA assay, with BSA as the standard.

To evaluate the broader utility of the AMAX system, we expressed five commercially important enzymes of different sizes and functions: T4 DNA ligase, T7 RNA polymerase, SUMO protease, HRV 3C protease, and D-hydantoinase (BsHase). Under standard induction conditions (0.1 mM IPTG, 37°C, and 3 hours), a small-scale 200 mL AMAX culture yielded substantial amounts of each enzyme: 7.5 mg of T4 ligase, 6.4 mg of T7 RNAP, 5.2 mg of SUMO protease, 13.8 mg of 3C protease, and 7.0 mg of BsHase (Fig. 6; Fig. S5). The rapid growth and high yields facilitate a streamlined process, allowing inoculation to purification to be completed within 8 hours, highlighting AMAX as an efficient and versatile platform for same-day protein production, offering practical advantages for both research and industrial applications.

## DISCUSSION

To achieve efficient and modular protein production, an ideal microbial chassis should possess several key traits: genetically tractable for easy modification, rapid growth, efficient utilization of low-cost carbon and nitrogen sources, and high stress tolerance for stable protein production (45–47). While some natural chassis cells exhibit some of these attributes, meeting all of these criteria simultaneously remains challenging. This study introduces AMAX, a high-performance, fast-growing chassis that combines complete compatibility with common existing vectors, enhanced protein production efficiency, phage resistance, and biosafety features. Specifically, AMAX outperformed BL21(DE3) in both growth rate and protein yield, producing sfGFP at levels constituting 60–70% of total cellular protein within 3 hours of induction. In contrast to Vmax, known for its fastest growth rate, AMAX grows similarly under routine lab conditions and does not require a specialized medium. Its broad salinity tolerance (1–3% salt) offers a potential advantage for industrial applications using seawater as a fermentation medium. Additionally, AMAX demonstrated minimal cytotoxicity in amoeba and HeLa cell models and retained susceptibility to clinically relevant antibiotics, underscoring its safety for biotechnological applications.

A key challenge for *E. coli* expression systems is bacteriophage contamination. Previous efforts to engineer phage-resistant strains have employed receptor modifications, CRISPR-Cas systems, and other anti-phage defense mechanisms (33, 36, 48). However, the evolutionary adaptability of phages often circumvents these defenses. Our study demonstrates an AMAX-BL21(DE3) mixed-strain expression setup expressing the same plasmid vector. Such a system exhibited robust production in the absence of phage, and the addition of *E. coli-*targeting phages did not affect the overall productivity in the mixture, indicating its effectiveness in mitigating phage-mediated lysis risk during large-scale production.

Transcriptional analyses revealed that AMAX shows global gene expression changes during growth-phase transitions under aerobic and anaerobic conditions. The activation of stress response pathways, including molecular chaperones and two-component systems, supports its adaptation to nutrient and environmental stresses. Interestingly, we noticed that genes in the TCA cycles were upregulated in the stationary phase in AMAX, which is in sharp contrast to that in *E. coli,* where it is downregulated (49). Considering the essential role of the TCA cycle in providing energy and building blocks to essential cellular activities, this upregulation may support the superior protein production capability of AMAX over *E. coli*. Additionally, the transcriptome data could guide further optimization of AMAX for specific application needs, including leveraging promoters of different strengths and ribosomal binding site (RBS) elements from highly expressed genes to develop tailored expression systems.

We have also characterized the proteomes associated with the OMVs derived from AMAX, which may be explored for potential biopharmaceutical applications, including vaccine development and protein delivery. We show that membrane proteins Pal and K1JB12 are capable of mediating the transport of cargo sfGFP into OMVs. Interestingly, GroEL and many other cytoplasmic proteins were found in AMAX-derived OMVs. This might result from the high sensitivity of our detection methods and various mechanisms that account for OMV production. For example, OMVs of *P. aeruginosa* may be generated through an explosive-lysis mode, in which the cellular membranes encapsulate released cytoplasmic contents (50). Nonetheless, the OMV of AMAX can be explored for encapsulating both cytosolic and membrane-bound proteins for future applications.

Compared to eukaryotic systems, prokaryotic expression platforms face limitations in posttranslational modifications and disulfide bond formation. Advances in co-expression systems, rare codon optimization, and synthetic biology tools have expanded the capabilities of prokaryotic systems, offering pathways for overcoming these challenges in AMAX (3, 4, 51). For example, to express proteins with multiple disulfide bonds, a more oxidizing cytoplasmic environment can be achieved by knocking out the *gor* and *trxB* reductase genes, and overexpression of the DsbC isomerase can enhance the production of correctly folded disulfide-bonded proteins (52, 53). For proteins requiring posttranslational modifications, a variety of posttranslational modifications—including phosphorylation, glycosylation, ubiquitination, methylation, myristoylation, and acetylation—have now been achieved in *E. coli* (54–59). For the production of toxic or membrane proteins, several strategies have been developed, such as utilizing low-copy plasmids, designing refined induction systems, mutating the T7 RNA polymerase promoter region, or secreting toxic proteins into the extracellular medium (60, 61). These successes pave the way for improving AMAX across diverse applications.

Emerging bacterial systems, such as *V. natriegens* and *P. putida*, have introduced novel capabilities in protein expression, excelling in traits such as rapid growth in *V. natriegens* and metabolic versatility in *P. putida* (5–9). Here, we show how AMAX uniquely combines fast growth (comparable to *V. natriegens* under lab conditions), high productivity (60–70% of cellular content), and compatibility with existing expression tools. Rather than presenting a paradigm shift in heterologous protein production, AMAX introduces a promising alternative or complementary system to *E. coli* or *V. natriegens*. It offers a powerful combination of fast growth (comparable to *V. natriegens* under lab conditions), high protein yield (60–70% of cellular content), salt tolerance (0 to 5% NaCl), and seamless co-culture with *E. coli* without compromising yields. Furthermore, AMAX does not require specialized conditions or media, simplifying its integration into existing workflows. While individually these traits may not be unique, their combination makes AMAX a practical and potentially valuable chassis for recombinant protein production in research and industrial settings.

## MATERIALS AND METHODS

### Strains and growth conditions

Strains, plasmids, and primers used in this study are described in Tables S4 to S6, respectively. All constructs were verified by sequencing. Unless stated otherwise, all strains employed in the study were cultivated aerobically at 37°C with agitation at 200 rpm in LB medium (1% tryptone [w/v], 0.5% yeast extract, and 0.5% NaCl). Of particular note, the WM6026 strain necessitated the supplementation of 2,6-diaminopimelic acid (Dap) to the culture medium at a final concentration of 100 µg/mL to sustain its normal growth. Antibiotics and inducers were used at the following concentrations: kanamycin (50 µg/mL), ampicillin (100 µg/mL), carbenicillin (100 µg/mL), chloramphenicol (25 µg/mL for *E. coli* and 2.5 µg/mL for AMAX), L-arabinose (0.1% or 0.01% [w/v] as indicated), and IPTG (0.1 mM or 1 mM as indicated).

### Bacterial genome sequencing

Genomic DNA was extracted using the TIANamp Bacteria DNA Kit (TIANGEN, #DP302). For library preparation, 2 µg of genomic DNA was subjected to end-repair and dA-tailing using the NEBNext Ultra II End Repair/dA-Tailing Module (New England Biolabs, #E7546S). The DNA was then cleaned with AMPure XP beads (Beckman Coulter, #A63882) and eluted in 31 µL of nuclease-free water.

Library construction followed the protocol of the Ligation Sequencing Kit 1D (Oxford Nanopore Technologies, #SQK-LSK109). The end-repaired DNA was ligated with AdaptomrMix using the Blunt/TA Ligation Master Mix (New England Biolabs, #M0367S). The ligated DNA was further purified with AMPure XP beads and the ABB buffer supplied in the kit. The concentration of the purified, ligated DNA was quantified using a Qubit 2.0 Fluorometer with the Qubit dsDNA HS Assay Kit (Thermo Fisher Scientific, #Q32851). Finally, the library was resuspended in 25 µL of elution buffer and loaded onto flow cells for sequencing.

Samples were sequenced individually on R9.4 flow cells (FLO-MIN106) for 24 hours each. Raw sequencing data in fast5 format, generated by MinKNOW, were base-called using Guppy (v4.2.2) to produce fastq files. Adapter sequences and redundant reads were trimmed and demultiplexed using PoreChop (v0.2.4) (https://github.com/rrwick/Porechop). Clean reads were then assembled with Unicycler (https://github.com/rrwick/Unicycler) to reconstruct the corresponding genomes (62).

### Preparation of electrocompetent cells

Overnight cultures were inoculated into fresh LB medium and allowed to grow until reaching an OD_600_ of 0.5–0.8. The culture was cooled on ice for 20–30 minutes, and then the cells were collected by centrifugation at 2,500 × *g* for 8 minutes at 4°C. The cell pellet was washed twice with pre-chilled 10% sucrose solution and finally resuspended in 10% sucrose solution (50 mL culture to 500 µL competent cells). A 50 µL of the cells was aliquoted into each tube and stored at −80°C for later use. A tube of electrocompetent cells was retrieved from −80°C and thawed on ice. The cells were then mixed with 200 ng of plasmid and transferred to a pre-chilled, clean electroporation cuvette. The electroporation was performed using a 2.5 kV, 2 cm cuvette program. The transformed cells were then added to 500 µL of fresh LB medium and incubated at 37°C for 1 hour. The culture was then spread on an LB plate containing the appropriate antibiotic, and the transformants were picked the next day.

### Determination of growth curve in flask

Overnight cultures were inoculated into fresh prewarmed LB media to make an initial OD_600_ ~0.03. To determine growth rates on bulk cultures, OD_600_ values of cultures growing at exponential phase were fit to an exponential function of form OD_600_ = Ae^Bt^, where *t* represents time, and *A* and *B* are constants. The fit parameters were used to determine doubling time with the formula: doubling time = ln (2)/*B*.

### Quantification of total protein and sfGFP yield in flask

Overnight strains harboring pET plasmids were freshly inoculated and grown to OD_600_ 0.6 ~ 0.8 in LB media with appropriate antibiotics. Then, 0.1 mM or 1 mM IPTG was added for protein induction at 37°C for 3 hours or at 20°C for 14 hours, respectively. After induction, cells were harvested at 4,500 *g* for 20 minutes. For every 50 mL initial culture, 5 mL PBS was added to resuspend the cell pellet. The resuspension was sonicated at 240 W for 15–20 minutes. Then, the supernatant was collected after two rounds of centrifugation at 12,000 × *g* for 20 minutes. The total protein of each sample was quantified using the Modified BCA Protein Assay Kit (NO. C503051, Sangon Biotech) by measuring the absorbance at 562 nm, with BSA as a standard. The sfGFP protein was quantified by GFP fluorescent quantification with excitation and emission spectrum of 480 nm and 510 nm, respectively. Purified sfGFP was used as the standard for this quantification, and its concentration was determined by the BCA assay.

### Determination of growth and sfGFP yield by microreader

Overnight cultures were sub-inoculated into black-welled, clear-bottom, 96-well sterile plates with an initial OD_600_ of approximately 0.05, with each well containing 100 µL of culture. The plates were first incubated for 2 hours at 37°C to allow for growth. Subsequently, 0.1 mM IPTG was added to each well to induce protein expression. Both the OD_600_ and sfGFP fluorescence (480 and 510 nm) were measured at 30-minute intervals using the BioTek Synergy H1 plate reader.

### Isolation of *E. coli* phage

The Dasha River water sample was first centrifuged at 4,000 × *g* for 10 minutes to remove any insoluble particles. The supernatant was then filtered through a 0.22 µm membrane to remove bacterial cells. To enrich the phage, 10 mL of the filtered supernatant was mixed with 10 mL of freshly cultured BL21(DE3) bacterial cells (OD_600_ ~0.6). This mixture was then incubated at 37°C for 48 hours, allowing the phages to propagate. After enrichment, the mixture was centrifuged at 4,000 × *g* for 10 minutes, and the resulting supernatant was filtered through a 0.22 µm membrane. The filtered solution was then plated onto a BL21(DE3) bacterial lawn to isolate potential *E. coli* phages.

### Extraction of phage genome and sequencing

Phages were concentrated to a titer of >10^9^ PFU/mL. A 200 µL sample of the phage culture was treated with DNase and RNase to remove any bacterial contaminations. Next, DNase was inactivated by heating the sample at 80°C for 15 minutes, while RNase was inactivated by adding a 150 mM EDTA solution and heating at 65°C for 15 minutes. The phage genome was then extracted using the TIANGEN Bacteria DNA Kit (#DP302) and sent to MEGIGENE company for genome sequencing. Briefly, the phage DNA was randomly sheared to generate fragments of the desired lengths, and the resulting sticky ends were repaired to create blunt ends. A single base “A” was then added to the 3′ end to allow for the connection of the DNA fragments to the special adapter with a 3′ end containing a “T” base. PCR was used to amplify the DNA fragments with the attached adapters, completing the construction of the entire library. The constructed and qualified library was used for cluster preparation and sequencing. Quality control was performed using the software Soapnuke, with clean reads aligned to a specified host genome using BWA software to remove any host sequences. Megahit software was used to assemble the high-quality reads from each sample into contigs. The assembled contigs were then compared to the CheckV virus database. The contigs of the target virus were checked for the formation of circular genomes, and if present, the overlapping regions were corrected and trimmed. The criteria for this correction were an overlap of more than 50 bp and an identity of more than 94%.

### Phage plaque assay

A 1 mL of fresh bacteria culture (with OD_600_ ~0.6) was spread onto plates to create a bacteria lawn. After air-drying for 20 minutes, a tenfold serial dilution of bacteriophage was added to the bacteria lawn. Following overnight cultivation, the number of plaques formed was counted.

### Phage infection during mixed fermentation

Overnight cultures were sub-inoculated into black-welled, clear-bottom, 96-well sterile plates with an initial OD_600_ of approximately 0.05, and each well contained 100 µL of culture. The plates were incubated for 2 hours at 37°C while shaking at 800 rpm. Subsequently, 0.1 mM IPTG was added to each well to induce protein expression for 1 hour. After that, phages (MOI = 0.1) were introduced to trigger *E. coli* lysis. Both the OD_600_ and sfGFP fluorescence (excitation at 480 nm and emission at 510 nm) were measured at 30-minute intervals.

### Protein purification using AMAX1

Overnight cultures were sub-inoculated into 200 mL of fresh LB medium supplemented with the appropriate antibiotics. When the cell density reached an OD600 of ~0.6, protein expression was induced by adding 0.1 mM IPTG, followed by incubation at 37°C for 3 hours. Cells were then harvested by centrifugation at 5,000 × *g* for 15 minutes.

For His-tagged proteins, the cell pellets were resuspended in lysis buffer (50 mM NaH_2_PO_4_, 300 mM NaCl, 5% glycerol, 20 mM imidazole, and pH 8.0) and lysed using high-pressure homogenization. The lysate was clarified by centrifugation at 15,000 × *g* for 40 minutes, and the supernatant was incubated with Ni–NTA (nickel–nitrilotriacetic acid) agarose beads for affinity purification. After washing, bound proteins were eluted using an imidazole gradient (50–400 mM) in the same buffer.

For GST-tagged proteins, the cell pellets were resuspended in lysis buffer (50 mM NaH_2_PO_4_, 300 mM NaCl, 5% glycerol, and pH 8.0) and lysed. Following centrifugation, the supernatant was incubated with glutathione agarose beads to capture GST-fusion proteins. The target proteins were eluted with a reduced glutathione gradient (5–10 mM).

### RNA extraction

Cells were retrieved from the exponential and stationary phase culture under aerobic and anaerobic conditions. Briefly, a 700 µL bacteria culture was mixed with 100 µL 8 × lysis buffer (8% [w/v] SDS and 16  mM EDTA) by vortex for 1  minute. Then, 800  µL prewarmed hot acidic phenol (65°C) was added and mixed by inverting immediately. Tubes were incubated at 65°C for 5  minutes, with brief mixing every 1  minute. After cooling for 10  minutes, the mixture was centrifuged at 13,000 *× g* for 2  minutes. The top supernatant was carefully transferred to a new tube and precipitated with 75% ethanol (final concentration) at −20°C for 1 hour. After precipitation, the pellet was harvested by centrifugation with 15,000 × *g* at 4°C for 15 minutes and washed twice with 75% ethanol. Then, the pellet was air-dried and resuspended with RNase-free water. To remove DNA, 2 µL of DNase I was added to each sample and incubated at 37°C for 30 minutes. The RNA was then precipitated, washed, and resuspended, just as previously mentioned.

### RNA-seq sequencing

The MEGIGENE platform was used to perform RNA sequencing. Initially, the total RNA was processed using a commercial ribosomal reagent kit to remove rRNA. Next, the obtained mRNA was converted into a library using the ALFA-SEQ RNA Library Prep Kit II. The first-strand cDNA was synthesized using reverse transcription with random hexamer primers, followed by the production of the second-strand cDNA using DNA polymerase I and RNase H. The resulting double-stranded cDNA was then end-repaired, had an A tail added to its 3' end, and was connected to a sequencing adapter. The library was then fragmented using magnetic beads and amplified by PCR. The size of the library was determined using the Qsep400 high-throughput nucleic acid protein analysis system (Hangzhou Houze Biotechnology Co., Ltd., China), and its concentration was measured using the Qubit 4.0 (Thermo Fisher Scientific, Waltham, USA). Paired-end reads of 150 bp were obtained by sequencing the library on either Illumina or MGI platforms.

### RNA-seq analysis

Total RNA sequencing was performed to explore gene expression profiles. Raw reads were processed with fastp (v0.23.4) to remove low-quality reads and adapter sequences, followed by rRNA removal using SortMeRNA (v4.3.4). Reads were aligned to the reference genome using STAR (v2.7.10) with default settings, and transcript-level TPM values were obtained with Salmon (v1.10.1) in alignment-free mode. Differential gene expression analysis was conducted using DESeq2 (v1.44.0) in R, with raw counts from Salmon as input. Normalization, dispersion estimation, and Wald tests identified differentially expressed genes, defined by an adjusted *P* value (pbadj) ≤0.05 (Benjamini-Hochberg correction) and |log_2_ Fold change| ≥ 1. Principal component analysis (PCA) was performed on VST-transformed data from DESeq2. Functional enrichment of differentially expressed genes was analyzed using ClusterProfiler (v4.12.3) for Gene Ontology (GO) and KEGG pathways, with significance at pbadj≤0.05. Data visualization was done with ggplot2 in R or through Bioinformatics.com.cn (accessed 10 Dec 2024).

### OMVs extraction and proteome analysis

Overnight cultures were transferred to 100 mL of fresh LB and incubated at 37°C with shaking at 200 rpm for 5 hours. The supernatants were collected by centrifuging at 4,500 × *g* for 20 minutes at 4°C and filtered through a 0.22 µm pore size filter to remove any remaining cells and debris. To prevent protein degradation, protease inhibitors (1 mM PMSF and 1 mM benzamidine) were added. The filtrates were then concentrated using an Amicon Ultra-4 centrifugal filter unit with a 100 kDa molecular weight cutoff, followed by ultracentrifugation at 200,000 × *g* for 1 hour at 4°C. The resulting pellet was washed with PBS and ultracentrifuged again at 200,000 × *g* for 1 hour at 4°C. The final OMV pellets were resuspended in 500 µL of PBS buffer and stored at −20°C. The proteome of the OMVs was then analyzed by the Shenzhen Academy of Metrology and Quality Inspection.

To test the recruitment of outer membrane proteins in OMVs, strains containing the pBAD construct plasmids were freshly grown to an OD_600_ of 0.6 and induced with 0.01% arabinose for 3 hours at 30°C. Samples of both whole cells and OMVs were collected and analyzed using SDS-PAGE and Western blotting.

### Fluorescence microscopy

For whole-cell bacteria, strains containing pBAD-Pal/K1JB12-sfGFP or sfGFP alone were cultured to the exponential phase and induced with 0.1% arabinose for 30 minutes. Cells were then pelleted, resuspended in PBS, and spotted onto the 1% agarose–0.5 ×PBS pad. For extracted OMV samples, OMVs were adjusted to approximately equal protein concentrations and spotted onto pads. Image acquisition was performed using a Zeiss 980 confocal microscope. All imaging experiments were conducted at least twice, and a representative result is shown.

### Amoeba plaque assay

Overnight strains were inoculated into fresh LB and grown to OD_600_ ~1. Cells were collected by centrifugation at 10,000 × *g* for 1  minute and resuspended in 1  mL PBS. A 300 µL volume of bacterial cells was plated on SM/five plates (2 g glucose, 2 g protease peptone, 0.2 g yeast extract, 0.2 g MgSO_4_·7H_2_O, 0.38 g KH_2_PO_4_, and 0.12 g K_2_HPO_4_/L; pH adjusted to 6.0–6.4) and allowed to dry under room temperature. *D. discoideum* cells were pelleted by centrifugation at 500 × *g* for 5  minutes, washed once with PBS, and resuspended in PBS to a final concentration of about 3  ×  10^6^ cells/mL. A series of fivefold dilutions of *D. discoideum* cells was plotted on the SM/five plates. The plaques were counted after the plates were incubated at 22°C for 3 to 7  days. The minimal number of *D. discoideum* cells deposited that was able to form plaque on the bacterial lawn was recorded.

### Cell toxicity assay

The HeLa cells were seeded in 48-well plates at a density of 2.5 × 10^5 cells per well in DMEM supplemented with 10% fetal bovine serum (FBS). Bacteria at varying MOIs were introduced into the wells and incubated for 2 hours. Post-infection, the cells were washed with PBS and exposed to DMEM containing 300 µg/mL gentamicin and 10% penicillin-streptomycin (PS) for 2 hours to eradicate any remaining extracellular bacteria. For quantifying intracellular bacteria, cells underwent three PBS washes before being lysed with 1% Triton X-100 at 37°C for 3 minutes. The lysates were then subjected to tenfold serial dilutions on LB plates to evaluate the survival of internalized bacteria. To assess cell death post-infection, cells were washed again following the elimination of extracellular bacteria and cultured in DMEM containing 10 µg/mL gentamicin to inhibit the growth of extracellular bacteria. Subsequently, propidium iodide (PI) (Abcam, ab14083) was added to the medium, and cell viability was monitored every hour using the IncuCyte system over a 24-hour period.

### Nematode infection assay

Different bacterial strains were inoculated into LB medium and incubated overnight at 37°C. The overnight cultures were then spread evenly onto 35-mm NGM agar plates, modified to contain 0.35% peptone instead of 0.25%. The plates were incubated at 37°C for 24 hours, followed by an additional incubation at 25°C for 16–24 hours, before seeding with synchronized L4-stage *C. elegans*. The slow-killing assay was conducted at 25°C, with animal survival monitored every 12 hours. To prevent progeny from hatching, 50 µg/mL 5-fluorodeoxyuridine (FUDR, Sigma, F0503) was added to the NGM. Each group included 60 worms, and worms were considered dead if they failed to respond to gentle touch.

### Antimicrobial susceptibility test

The antimicrobial susceptibility assay was conducted using the BD Phoenix AST system at the Shenzhen Third People’s Hospital. The types of antibiotics and their minimum inhibitory concentrations (MICs) were determined using the CLSI M45 ED3 2016 guidelines.

## Data Availability

The transcriptome data have been deposited in the National Microbiology Data Center (NMDC) (https://nmdc.cn/resource/genomics/project/detail/NMDC10019701). Data are accessible under the identifiers NMDC40080152 (Aero.exp), NMDC40080153 (Aero.sta), NMDC40080154 (Ana.exp), and NMDC40080155 (Ana.sta). The strain is available for scientific research upon request. Data supporting the findings of this study are available within the paper or from the corresponding author upon request.

## SUPPLEMENTAL MATERIAL

Supplemental material aem.00780-25-s0001.pdf Figures S1 to S5; Tables S1 to S6.

ASM does not own the copyrights to Supplemental Material that may be linked to, or accessed through, an article. The authors have granted ASM a non-exclusive, world-wide license to publish the Supplemental Material files. Please contact the corresponding author directly for reuse.
