# Supplementary material for: Aeromonas spp. as a fast-growing high-performance chassis for protein production
Source: Appl Environ Microbiol. 2025 Jun 3;91(7):e00780-25. doi: 10.1128/aem.00780-25 (PMC12285260; doi:10.1128/aem.00780-25)
Supplement: Supplemental material — Figures S1 to S5; Tables S1 to S6. [file aem.00780-25-s0001.pdf]

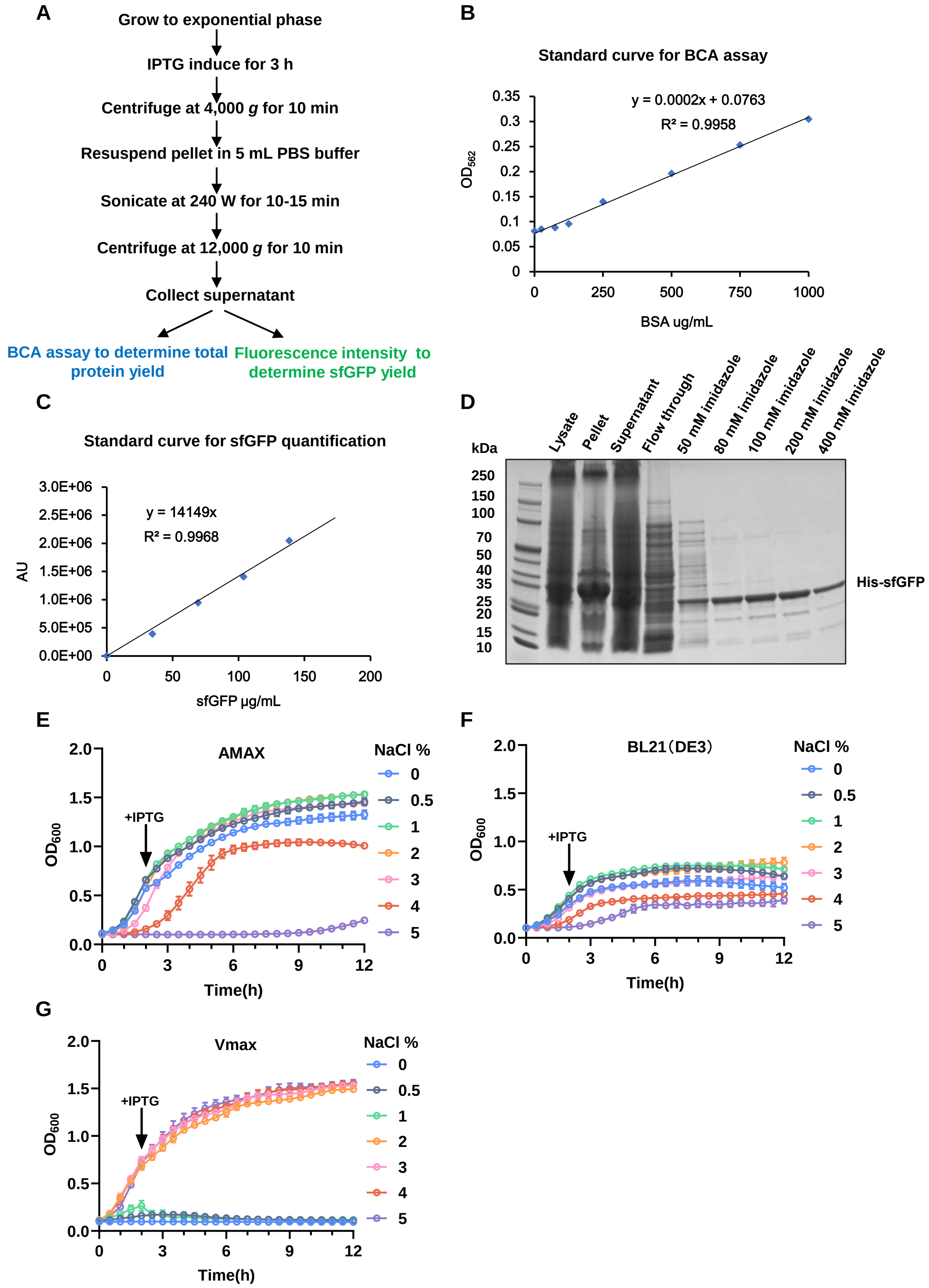

Figure S1. AMAX demonstrates robust growth and protein yield across various salinity conditions. (A) Flowchart for determining the total protein and sfGFP yields shown in Figure 1C. (B) Standard curve for determining total protein yield, using BSA as the standard. (C) Standard curve for

determining sfGFP fluorescence intensity, using purified sfGFP as the standard. (D) Coomassie-stained gel of purified sfGFP. (E-G) Growth of AMAX (E), BL21(DE3) (F), and Vmax (G) in LB medium under varying NaCl concentrations, measured by OD_{600} using a 96-well microplate reader.

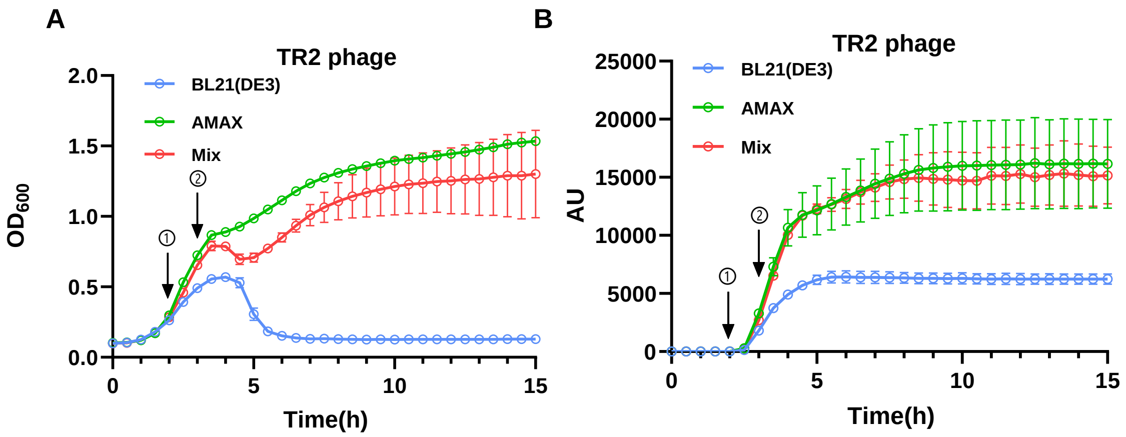

Figure S2. Performance comparison of single-strain and mixed-strain expression systems under phage infection. (A-B) Growth (A) and protein expression (B) of the three systems with TR2 phage infection.

A

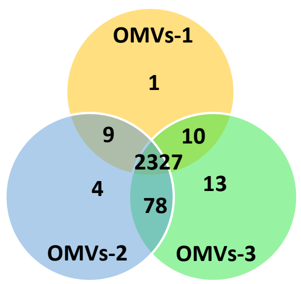

B

	Uniport ID	Description	Mean±SD (*10^7)
1	K1JNX6	Bacteriophage Mu GpT domain-containing protein	95.3±8.3
2	K1JH43	p2 family phage major capsid protein	77.9±7.9
3	K1K4V0	DUF3103 domain-containing protein	69.2±32.2
4	K1JQB9	Extracellular exonuclease ExeM, a biofilm formation-associated exonuclease that cleaves extracellular DNA	68.2±24.5
5	K1K1B7	Chaperone GroL	44.9±19.5
6	K1JBK7	Outer membrane porin OmpC/OmpF/PhoE	34.7±2.1
7	K1K8V0	Outer membrane protein OmpK	33.0±0.9
8	K1JR94	OmpA-like domain-containing protein	32.5±2.2
9	K1JK89	Uncharacterized protein	31.5±1.6
10	K1JKN9	TIGR04219 family outer membrane beta-barrel protein	30.1±4.7

C

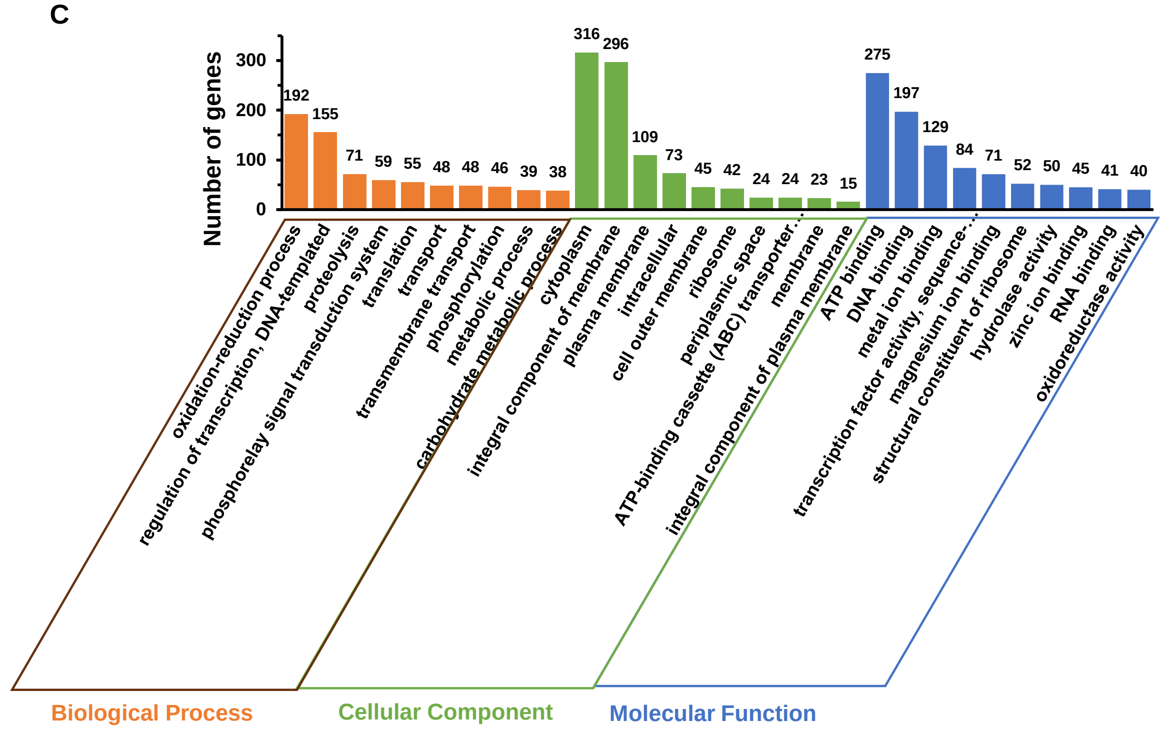

Figure S3. Extraction and characterization of OMVs. (A) Venn diagram illustrating the overlap of proteins identified across three replicates. (B) List of the top 10 most abundant proteins found in the OMVs. (C) GO enrichment analysis of proteins present in OMVs.

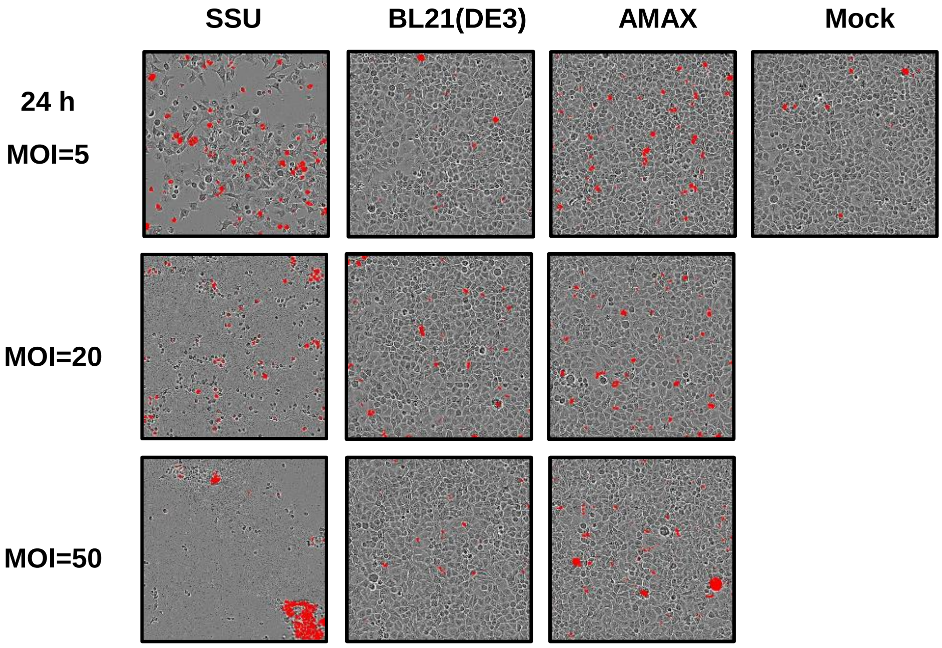

Figure S4. Microscopy of Hela cell morphology post-infection with AMAX at different MOIs. HeLa cells were infected with AMAX at various MOIs for 2 hours, followed by treatment with gentamicin to eliminate extracellular bacteria. Cell death was assessed by adding Propidium Iodide (PI) and monitoring cell viability for 24 hours using the IncuCyte system. Representative images show cell morphology and viability at different MOIs.

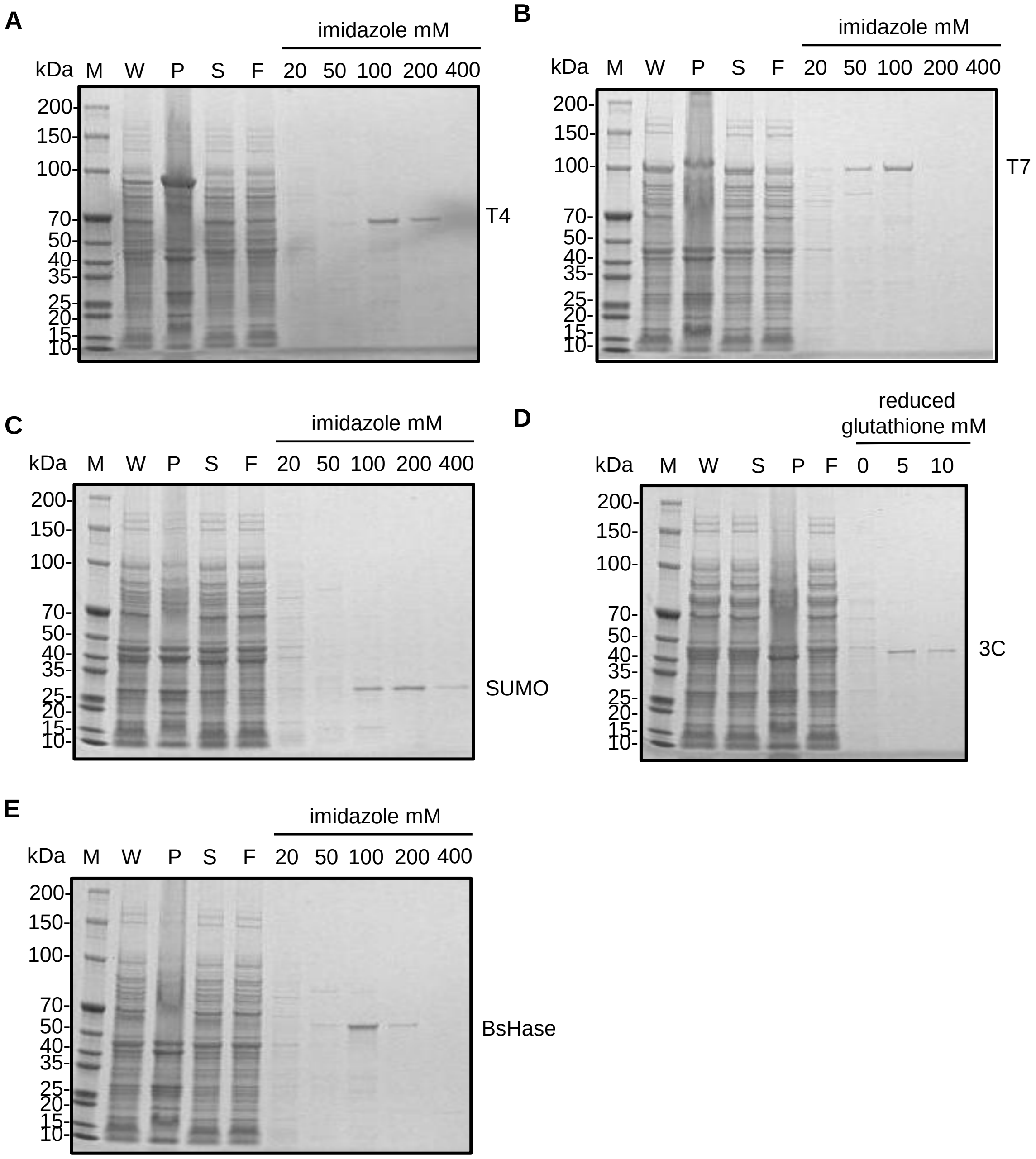

Figure S5. Purification of different enzymes using AMAX. Purification of His-T4 ligase (A), T7 RNAP-His (B), His-SUMO protease (C), GST-HRV 3C protease (D), and BsHase-His (E) from AMAX cultures. Proteins were expressed by induction with 0.1 mM IPTG for 3 hours at 37°C. After harvesting the cells, proteins were purified using either Ni-NTA or glutathione agarose beads, depending on the tag (His or GST). The following fractions were collected and analyzed: W, whole cell; P, cell pellet; S, supernatant; F, flow-through. Elution was achieved using imidazole or glutathione gradients. Supernatants and eluted samples of these enzymes are compared in Figure 6.

Table S1. List of genes knocked out in the AMAX

Name	Function
alt	heat-labile enterotoxin
ast	heat-stable enterotoxin
act	enterotoxin
hlyA	hemolysin
ahh1	extracellular hemolysin
T6SS	type six secretion system
T3SS	type three secretion system
vapA	S-layer protein
flrA	polar flagella transcription regulator
lafK	lateral flagella transcription regulator
blaOXA	Class D penicillinases
blaAmpC	Class C cephalosporinases

Table S2. Vectors compatible in AMAX

Plasmid	Ori	Promoter	Inducer	Selection marker
pET28a	pMB1	T7	IPTG	Kanamycin
pBAD24	pBR322	Para	Arabinose	Ampicillin
p15A	p15A	Ptet	Anhydrotetracycline	Chloramphenicol
pPSV37	pBR322	PlacUV5	IPTG	Gentamicin

Table S3. Two-component systems in AMAX

No.	Name	Function
1	PhoR-PhoB	PO_4^{3-}starvation
2	PhoQ-PhoP	Mg^{2+} transport
3	EnvZ-OmpR	Osmotic stress
4	RstB-RstA	Stress
5	CpxA-CpxR	Envelope stress
6	CreC-CreB	PO^{3-} regulation
7	QseC-QseB	Quorum sensing
8	KdpD-KdpE	K^+ transport
9	ArcB-ArcA	Anoxic redox control
10	NarQ-NarP	NO_2 respiration
11	UhpB-UhpA	Hexose phosphates uptake
12	BarA-UvrY	Central-carbon metabolism
13	GlnL-GlnG	N_2 regulation
14	GlrK-GlrR	Amino sugar metabolism
15	PgtB-PgtA	Phosphoglycerate transport
16	FlrB-FlrC	Polar flagellar synthesis
17	TorS-TorR	TMAO respiration

Table S4. Strains used in this study.

Strain	Genotype	Description	Source
E. coli	pir1	Strain used for cloning	Lab stock
	WM6026	Strain used for conjugation	Lab stock
	DH5alpha	Strain used for cloning	Lab stock
	T-fast	Strain used for cloning	Lab stock
	BL21(DE3)	Strain used for protein expression	Lab stock
Aeromonas	*Aeromonas dhakensis* SSU	Pathogenic control in biosafety assay	Lab stock
	TD1418	Lab collection, parental of AMAX	This study
	AMAX		This study
	Aeromonas sp. 2932	Environmental isolate	This study
	Aeromonas sp. 2934	Environmental isolate	This study
	Aeromonas sp. 4008	Environmental isolate	This study
	Aeromonas sp. 4009	Environmental isolate	This study

Table S5. Plasmids used in this study.

	Plasmid	Description	Source
pDS132		Suicidal conjugation vector for all chromosomal allelic changes	(1)
	dtype I fimbriae	Suicidal vector to construct AMAX chromosomal Δ*type I fimbriae*	This study
pBAD	pBAD24kan	Arabinose inducible expression plasmid, kanamycin resistance	Lab stock
	pBAD24kan-Pal-3V5	Arabinose inducible plasmid to express Pal-3V5	This study
	pBAD24kan-LamB-3V5	Arabinose inducible plasmid to express LamV-3V5	This study
	pBAD24kan-K1JB12-3V5	Arabinose inducible plasmid to express K1JB12-3V5	This study
	pBAD24kan-OmpA-3V5	Arabinose inducible plasmid to express OmpA-3V5	This study
	pBAD24kan-Pal-sfgfp	Arabinose inducible plasmid to express Pal-sfGFP	This study
	pBAD24kan-K1JB12-sfgfp	Arabinose inducible plasmid to express K1JB12-sfGFP	This study
pET	pET28a-sfGFP-6His	Plasmid to express sfGFP	Lab stock

psf1877 Ulp1 SUMO	Plasmid to express SUMO protease Ulp1	Lab stock
pXL1144 GST-HRV3C	Plasmid to express 3HRV C protease	Lab stock
pET28a-T4 Ligase	Plasmid to express T4 ligase	Lab stock
pET22b-T7 RNAP-6His	Plasmid to express T7 RNA polymerase	This study
pET22b-BsHase-AHT-6his	Plasmid to express BsHase	This study

Table S6. Primers used in this study.

Primer	Sequence (5’-3’)	Description
pDS132-hifi-f	cgatccttttaacccatcac	Forward primer to amplify pDS132 vector
pDS132-hifi-r	cttctagaggtaccgcatgc	Reverse primer to amplify pDS132 vector
pDS132-f	tgttgcatgggcataaagttgc	Forward confirmation primer of pDS132 vector
pDS132-r	acggctgacatgggaattcc	Reverse confirmation primer of pDS132 vector
type I fimbriae-KO1	gtgatgggttaaaaaggatcggat cgctatttcagcccgca	Forward primer to amplify the upstream homologs arm for constructing in-frame deletion of Type I fimbriae
type I fimbriae-KO2	atcgttgcctatgaataaggtcag a	Reverse primer to amplify the upstream homologs arm for constructing in-frame deletion of Type I fimbriae
type I fimbriae-KO3	ccttattcataggcaacgattttgtt agctttcattcgtttatacct	Forward primer to amplify the downstream homologs arm for constructing in-frame deletion of Type I fimbriae
type I fimbriae-KO4	gcatgcggtacctctagaagtggt gcttcccttcacaagt	Reverse primer to amplify the downstream homologs arm for constructing in-frame deletion of Type I fimbriae
type I fimbriae-KO5	tatcttctgcgcccccattg	Forward confirmation primer of Type I fimbriae deletion
type I fimbriae-KO6	tcagcatgaccttgggagtg	Reverse confirmation primer of Type I fimbriae deletion
dAct-KO1	gtgatgggttaaaaaggatcgtgc cgcgaggatgattgac	Forward primer to amplify the upstream homologs arm for constructing in-frame deletion of Act
dAct-KO2	gccggatatgatcaatgacaag	Reverse primer to amplify the upstream homologs arm for constructing in-frame deletion of Act
dAct-KO3	tgtcattgatcatatccggcgcag ccaatcaataacggca	Forward primer to amplify the downstream homologs arm for constructing in-frame deletion of Act
dAct-KO4	gcatgcggtacctctagaagttcc agcgcttcggtgtc	Reverse primer to amplify the downstream homologs arm for constructing in-frame deletion of Act
dAct-KO5	ggcggatctggggttatgtc	Forward confirmation primer of Act deletion
dAct-KO6	aaattccatctccggggtcac	Reverse confirmation primer of Act deletion
dAlt-KO1	gtgatgggttaaaaaggatcgcc gcttacctacatccctgt	Forward primer to amplify the upstream homologs arm for constructing in-frame deletion of Alt

dAlt-KO2	ctggcttactgcttgagcagcagc atagcactgacgactg	Reverse primer to amplify the upstream homologs arm for constructing in-frame deletion of Alt
dAlt-KO3	ctgctcaagcagtaagccag	Forward primer to amplify the downstream homologs arm for constructing in-frame deletion of Alt
dAlt-KO4	gcatgcggtacctctagaagagct gacccagtcggagttg	Reverse primer to amplify the downstream homologs arm for constructing in-frame deletion of Alt
dAlt-KO5	gtgttcttctactggggcgt	Forward confirmation primer of Alt deletion
dAlt-KO6	actatctctccaccctcggc	Reverse confirmation primer of Alt deletion
dahh1-KO1	gtgatgggttaaaaaggatcgca gcagcaacttgcaatccat	Forward primer to amplify the upstream homologs arm for constructing in-frame deletion of Ahh1
dahh1-KO2	agcgggagcttgcgtgat	Reverse primer to amplify the upstream homologs arm for constructing in-frame deletion of Ahh1
dahh1-KO3	tcatcacgcaagctcccgctccg gccaaccactgaca	Forward primer to amplify the downstream homologs arm for constructing in-frame deletion of Ahh1
dahh1-KO4	gcatgcggtacctctagaagttcc tatgcaacgttggccc	Reverse primer to amplify the downstream homologs arm for constructing in-frame deletion of Ahh1
dahh1-KO5	cataaacggggtttggggct	Forward confirmation primer of Ahh1 deletion
dahh1-KO6	agcacaagaacttcaaccgc	Reverse confirmation primer of Ahh1 deletion
dHlyA-KO1	gtgatgggttaaaaaggatcgcc atgccgccatccagta	Forward primer to amplify the upstream homologs arm for constructing in-frame deletion of HlyA
dHlyA-KO2	tcctgatgctgctggttgccgctca ggaatcgtaaaaggagct	Reverse primer to amplify the upstream homologs arm for constructing in-frame deletion of HlyA
dHlyA-KO3	ggcaaccagcagcatcag	Forward primer to amplify the downstream homologs arm for constructing in-frame deletion of HlyA
dHlyA-KO4	gcatgcggtacctctagaagaag gcatactcggagcatcaag	Reverse primer to amplify the downstream homologs arm for constructing in-frame deletion of HlyA
dHlyA-KO5	taggaaccaaacagcggcg	Forward confirmation primer of HlyA deletion
dHlyA-KO6	tcgtcacccacaacatgcaa	Reverse confirmation primer of HlyA deletion
dAst-KO1	gtgatgggttaaaaaggatcgac aacctggagaccgtcgag	Forward primer to amplify the upstream homologs arm for constructing in-frame deletion of Ast
dAst-KO2	ggtacgtgcgtgcatggtt	Reverse primer to amplify the upstream homologs arm for constructing in-frame deletion of Ast
dAst-KO3	caaccatgcacgcacgtaccgtg aaaaagtcctgacccagac	Forward primer to amplify the downstream homologs arm for constructing in-frame deletion of Ast
dAst-KO4	gcatgcggtacctctagaagagt gaagagcttcaaggagtcg	Reverse primer to amplify the downstream homologs arm for constructing in-frame deletion of Ast
dAst-KO5	atctcgtcccacgaaatgctt	Forward confirmation primer of Ast deletion
dAst-KO6	tgtttttaaagcgcagaggcg	Reverse confirmation primer of Ast deletion

dlafK-KO1	gtgatgggttaaaaaggatcgac caaggatcctgtcttgctg	Forward primer to amplify the upstream homologs arm for constructing in-frame deletion of LafK
dlafK-KO2	atcccttagttttgaagctgtaaatc cgaaagcataagaacccga	Reverse primer to amplify the upstream homologs arm for constructing in-frame deletion of LafK
dlafK-KO3	cagcttcaaaactaagggataaac c	Forward primer to amplify the downstream homologs arm for constructing in-frame deletion of LafK
dlafK-KO4	gcatgcggtacctctagaagcgc atccagtgaaatgatggt	Reverse primer to amplify the downstream homologs arm for constructing in-frame deletion of LafK
dlafK-KO5	ggaagagctaaacctggcgt	Forward confirmation primer of LafK deletion
dlafK-KO6	cgcactgggcttctcttcat	Reverse confirmation primer of LafK deletion
dFlrA-KO1	gtgatgggttaaaaaggatcgac gtggactatctgctctgc	Forward primer to amplify the upstream homologs arm for constructing in-frame deletion of FllrA
dFlrA-KO2	gacccttatgcttccttgcttcccat tgccatcatatccatcc	Reverse primer to amplify the upstream homologs arm for constructing in-frame deletion of FllrA
dFlrA-KO3	agcaaggaagcataagggtca	Forward primer to amplify the downstream homologs arm for constructing in-frame deletion of FllrA
dFlrA-KO4	gcatgcggtacctctagaaggttg gcgatgaggttgttgac	Reverse primer to amplify the downstream homologs arm for constructing in-frame deletion of FllrA
dFlrA-KO5	gggatgggcaagagcaagtg	Forward confirmation primer of FllrA deletion
dFlrA-KO6	gcgggtggtaaagaagggt	Reverse confirmation primer of FllrA deletion
dCphA-KO1	gtgatgggttaaaaaggatcgcc ggcagtttttggaacagag	Forward primer to amplify the upstream homologs arm for constructing in-frame deletion of Bla CphA
dCphA-KO2	ccagcttatgactggggcaccca gaagctcgccatcag	Reverse primer to amplify the upstream homologs arm for constructing in-frame deletion of Bla CphA
dCphA-KO3	gtgccccagtcataagctg	Forward primer to amplify the downstream homologs arm for constructing in-frame deletion of Bla CphA
dCphA-KO4	gcatgcggtacctctagaagcctc gagggatgcggtaaag	Reverse primer to amplify the downstream homologs arm for constructing in-frame deletion of Bla CphA
dCphA-KO5	ccgggttaacgaacttgctg	Forward confirmation primer of Bla CphA deletion
dCphA-KO6	caccacctgaaaatcccca	Reverse confirmation primer of Bla CphA deletion
dAmpC-KO1	gtgatgggttaaaaaggatcgcat cggcgaggtggatctg	Forward primer to amplify the upstream homologs arm for constructing in-frame deletion of Bla AmpC
dAmpC-KO2	cagaccggtccacatcaaag	Reverse primer to amplify the upstream homologs arm for constructing in-frame deletion of Bla AmpC
dAmpC-KO3	ctttgatgtggaccggtctgcacg ccatcctgagcaag	Forward primer to amplify the downstream homologs arm for constructing in-frame deletion of Bla AmpC

dAmpC-KO4	gcatgcggtacctctagaagttgc gccgtttgttcaaaga	Reverse primer to amplify the downstream homologs arm for constructing in-frame deletion of Bla AmpC
SdAmpC-KO5	ccagcaggtagtcgagggtg	Forward confirmation primer of Bla AmpC deletion
dAmpC-KO6	gacgattaaccgtgagcgga	Reverse confirmation primer of Bla AmpC deletion
dT6SS-KO1	gtgatgggttaaaaaggatcggc ctgatgcaaaaagccgtt	Forward primer to amplify the upstream homologs arm for constructing in-frame deletion of T6SS
dT6SS-KO2	acatggagttggcatggaattg	Reverse primer to amplify the upstream homologs arm for constructing in-frame deletion of T6SS
dT6SS-KO3	attccatgccaactccatgtgaaa gatatggttaagagtttggcaagg	Forward primer to amplify the downstream homologs arm for constructing in-frame deletion of T6SS
dT6SS-KO4	gcatgcggtacctctagaagttttc ggtgattagcgcagc	Reverse primer to amplify the downstream homologs arm for constructing in-frame deletion of T6SS
dT6SS-KO5	gcctgatgcaaaaagccgtt	Forward confirmation primer of T6SS deletion
dT6SS-KO6	gctttcccgcttgggtatga	Reverse confirmation primer of T6SS deletion
dT3SS-KO1	tgatgggttaaaaaggatcggtcg gattgatgctgctctg	Forward primer to amplify the upstream homologs arm for constructing in-frame deletion of T3SS
dT3SS-KO2	gaagcacgctcatagcacaca	Reverse primer to amplify the upstream homologs arm for constructing in-frame deletion of T3SS
dT3SS-KO3	gtgtgctatgagcgtgcttcggct cgctctggtaaacgc	Forward primer to amplify the downstream homologs arm for constructing in-frame deletion of T3SS
dT3SS-KO4	gcatgcggtacctctagaaggcc agtacttcgatccgctg	Reverse primer to amplify the downstream homologs arm for constructing in-frame deletion of T3SS
dT3SS-KO5	tgctggacagaatcggcaa	Forward confirmation primer of T3SS deletion
dT3SS-KO6	ccaaggatcgataaccttggca	Reverse confirmation primer of T3SS deletion
dvapA-KO1	tgatgggttaaaaaggatcgacc gaaagagacaaccccctc	Forward primer to amplify the upstream homologs arm for constructing in-frame deletion of VapA
dvapA-KO2	gcgttcatggtaatcagtgtcttttt cattagaatcattc	Reverse primer to amplify the upstream homologs arm for constructing in-frame deletion of VapA
dvapA-KO3	acactgattaccatgaacgcattct aatcgtt	Forward primer to amplify the downstream homologs arm for constructing in-frame deletion of VapA
dvapA-KO4	gcatgcggtacctctagaaggga tggtgctgatcacccc	Reverse primer to amplify the downstream homologs arm for constructing in-frame deletion of VapA
dvapA-KO5	cactgaagcgtcgctttttacc	Forward confirmation primer of VapA deletion
dvapA-KO6	cagggtagaaaattggcgagag g	Reverse confirmation primer of VapA deletion
pBAD24-V5-hifi-f	ggtaaacctattcctaatcctctcct t	Forward primer to amplify pBAD24-3V5 vector
pBAD24-hifi-r	ggtacctcctgctagcccaaa	Reverse primer to amplify pBAD24-3V5 vector

pBAD-f	agtccacattgattatttgcacgg	Forward confirmation primer of pBAD vector
pBAD-r	ttcacttctgagttcggcatgg	Reverse confirmation primer of pBAD vector
pBAD24-LamB-3V5-f	ttgggctagcaggaggtaccatgaaagcaaagtggctccc	Forward primer to amplify LamB
pBAD24-LamB-3V5-r	ggattaggaataggtttaccccaccaagcttcagcttgaac	Reverse primer to amplify LamB
pBAD24-OmpA-3V5-f	ttgggctagcaggaggtaccatgaaaatggctccttccctgat	Forward primer to amplify OmpA
pBAD24-OmpA-3V5-r	ggattaggaataggtttacccttctgaacttcttgtacgccaga	Reverse primer to amplify OmpA
pBAD24-K1JB12-3V5-f	ttgggctagcaggaggtaccatgaaatttggcatcaaatccgttg	Forward primer to amplify K1JB12
pBAD24-K1JB12-3V5-r	ggattaggaataggtttaccccaaccttcaccaggcc	Reverse primer to amplify K1JB12
pBAD24-Pal-3V5-f	ttgggctagcaggaggtaccatgcaactcaataaactgctcaag	Forward primer to amplify Pal
pBAD24-Pal-3V5-r	ggattaggaataggtttaccgtaaaccaggaccgcacgg	Reverse primer to amplify Pal
K1JB12-sfGFP-r	ccaccgccgccagaaccgccccaacctttcaccaggcc	Reverse primer to amplify K1JB12 to construct pBAD-K1JB12-sfGFP
Pal-sfGFP-r	ccaccgccgccagaaccgccgtaaaccaggaccgcacgg	Reverse primer to amplify Pal to construct pBAD-Pal-sfGFP
pET-hifi-f	aagcttgcggccgcactcga	Forward primer to amplify pET22b-Chis vector
pET-hifi-r	catatgtatatctccttcttaaagttaaacaaaattatttctagag	Reverse primer to amplify pET22b-Chis vector
pETDuet-f	cacgatgcgtccggcgtagagg	Forward confirmation primer of pET vector
pETDuet-r	ggttatgctagttattgctcagcggt	Reverse confirmation primer of pET vector
T7 RNAP-f	aagaaggagatatacatatgaacacgattaacatcgctaagaacg	Forward primer to amplify T7 RNAP
T7 RNAP-r	tcgagtgcggccgcaagcttcgcgaacgcgaagtccga	Reverse primer to amplify T7 RNAP
BsHase-f	aagaaggagatatacatatgatgaccaaaattattaaaaatggtaccatcg	Forward primer to amplify BsHase
BsHase-r	tcgagtgcggccgcaagcttaatggtcagttcttcgctctgt	Reverse primer to amplify BsHase

References

1. Philippe N, Alcaraz JP, Coursange E, Geiselmann J, Schneider D. 2004. Improvement of pCVD442, a suicide plasmid for gene allele exchange in bacteria. Plasmid 51:246–255.
